# Can single disease payment impact hospitalization expenses and quality in district hospital? A case study in Fujian, China

**DOI:** 10.1186/s12939-024-02134-2

**Published:** 2024-03-13

**Authors:** Liangwen Zhang, Wanqiu Sha, Qiyu Lin, Ya Fang

**Affiliations:** 1 State Key Laboratory of Molecular Vaccinology and Molecular Diagnostics, School of Public Health, Xiamen University, Xiamen Fujian, 361102 PR China; 2 Key Laboratory of Health Technology Assessment of Fujian Province University, School of Public Health, Xiamen University, Xiamen Fujian, 361102 PR China

**Keywords:** District hospitals, Single disease payment, Effect evaluation, Medical expenses, China

## Abstract

**Background:**

China is exploring payment reform methods for patients to address the escalating issue of increasing medical costs. While most district hospitals were still in the stage of Single Disease Payment (SDP) due to conditions, there is a scarcity of research on comprehensive assessment of SDP. This study aims to evaluate the implementation of SDP in a district hospital, and provided data support and scientific reference for improving SDP method and accelerating medical insurance payment reform at district hospitals.

**Methods:**

Data was collected from 2337 inpatient medical records at a district hospital in Fuzhou, China from 2016 to 2021. These diagnoses principally included type 2 diabetes, planned cesarean sections, and lacunar infarction. Structural variation analysis was conducted to examine changes in the internal cost structure and dynamic shifts in medical expenses for both the insured (treatment group) and uninsured (control group) patients, pre- and post-implementation of the SDP policy on August 1, 2018. The difference-in-differences (DID) method was employed to assess changes in hospitalization expenses and quality indicators pre- and post-implementation. Furthermore, subjective evaluation of medical quality was enhanced through questionnaire surveys with 181 patients and 138 medical staff members.

**Results:**

The implementation of SDP decreased the medical expenses decreased significantly (*P <* 0.05), which can also optimize the cost structure. The drug cost ratio descended significantly, and the proportion of laboratory fee rose slightly. The changes in infection rate, cure rate, and length of stay indicated enhanced medical quality (*P <* 0.05). The satisfaction of inpatients with SDP was high (89.2%). Medical staff expressed an upper middle level of satisfaction (77.2%) but identified difficulties with the implementation such as “insufficient coverage of disease types”.

**Conclusion:**

After the implementation of SDP in district hospitals, considerable progress has been achieved in restraining medical expenses, coupled with notable enhancements in both medical quality and patient satisfaction levels. However, challenges persist regarding cost structure optimization and underutilization of medical resources. This study suggests that district hospitals can expedite insurance payment reform by optimizing drug procurement policies, sharing examination information, and strengthening the management of medical records.

**Supplementary Information:**

The online version contains supplementary material available at 10.1186/s12939-024-02134-2.

## Background

The escalating global burden of medical expenses has emerged as a shared concern among patients, payers, and policymakers worldwide [1]. This issue is particularly acute in China, the world’s largest developing country, where several factors contribute to the escalating costs. These include rapid urbanization, increased demand for medical services, expanded medical insurance coverage, and the prevalence of diseases linked to an aging population. According to *the Statistical Bulletin on the Development of National Medical Security Undertakings in 2022*, total expenditure from China’s national basic medical insurance fund reached 309.22 billion *yuan*, marking a year-on-year increase of 7.6% [2]. In the context of ongoing medical insurance reform, this upward trend in expenses underscores the urgent need for efficient resource allocation and effective cost control measures in the operation of medical insurance funds.

It is widely acknowledged that different payment systems lead to distinct behaviors of service providers and payers [3, 4]. The payment methods for medical insurance can be categorized into pre-payment and post-payment systems in chronological order. Pre-payment methods include capitation, disease-based payment, Diagnosis Related Groups (DRGs) payment and Diagnosis Intervention Packet (DIP) payment [5, 6]. Post-payment methods mainly involve paying for services rendered or hospitalization bed days. To address the issues of over-examination, over-treatment, and drug abuse caused by fee-for-service payments, China has been exploring diversified medical insurance reimbursement models. In China, notable regional economic disparities have prompted certain areas to pioneer the adoption and modification of foreign payment models like DRGs and DIP. Research shows these advanced methods, which account for disease complexity, are pivotal in managing medical expenses [7], reducing patients’ financial strain, and standardizing clinical diagnostics and treatment. However, due to various constraints, many regions still use SDP [8]. SDP calculates the total cost for treating a specific disease, ignoring comorbidities and complications, and sets a standard payment rate. Hospitals bill patients with the disease at this rate. It is often implemented in the initial stages of service item charge reform and is applied to some diseases with clear diagnoses for billing purposes.

According to official data from the *China Health Statistical Yearbook 2022*, as of the end of 2021, district hospitals accounted for 47.29% (17,294) of the country’s total hospitals (36,570) [9]. District hospitals, primarily treating common and frequently-occurring diseases, often encounter challenges in advancing medical insurance payment reform due to limitations in information infrastructure, case management capabilities, medical personnel awareness, and the quality of medical records. These limitations render them a weak link in China’s medical insurance system. Taking Fujian Province as an example, district-level hospitals began to adopt and implement DRGs in 2017. However, the majority still remain at the stage of implementing SDP [10]. Therefore, evaluating the effect of current SDP implementation can help identify problems and put forward measures while also laying a foundation for future transitions towards more refined payment methods.

Reforming the Medicare payment model is a contentious issue in healthcare reform due to rapidly increasing costs, impacting both medical care expenses and service delivery by doctors. Clarifying the process and impact of existing healthcare payment reform is of significant referential value for countries currently undergoing such reforms. In 1983, the United States pioneered a pre-payment system based on DRG-PPS for inpatient services [11], which has since been studied and implemented with varying characteristics in many countries. The United States expanded their application to include outpatient care, home care, and other areas [12]. However, excessive fee regulation led to issues regarding neglect of quality of care. This led to the introduction of a value-based payment method, aiming to ensure quality within the DRG framework.

Australia introduced the AN-DRG system for indigenous patients in 1992, which was subsequently updated to AR-DRG in 1998 [13]. This system effectively manages hospital costs and prevents excessive expenses, curbing medical expenditures and discouraging unnecessary testing and medication. In 2000, Germany introduced the G-DRG system, which necessitated hospitals to seek approval for novel technologies and projects [14]. This ensured that reimbursement was predicated on meticulous coding and accounted for cost variations arising from innovations. The implementation of DRGs reduced medical expenses alongside improved overall hospital service efficiency.

The development and implementation of the DRG-PPS model in other countries, especially in the United States, have provided critical insights for China’s healthcare payment reform initiatives. China has been transitioning its medical insurance payment approach from a fee-for-service model to a disease-based payment system. Notably, the practical application of DRG-based payments was initiated in Beijing as early as 1988. However, the full adoption of the DRG payment system in China has faced challenges due to the limitations in the early stages of the country’s medical management system and the level of informatization. These factors have so far hindered the comprehensive implementation of the DRG model in China [15]. To alleviate pressure on medical insurance funds and control growth of healthcare expenses, the concept of SDP emerged. As a response to the challenges encountered with DRG payments, SDP method is applied in cases of common and frequently occurring diseases to establish payment limits.

As medical insurance payment methods are being reformed nationwide, some cities have started experimenting with diversified methods like DRGs and DIP payments, which have yielded positive results. In 2018, Sanming City in Fujian Province switched from SDP to C-DRG payments, and significantly relieved financial burdens for patients with lower illness severities [16]. In 2022, a case in Guangzhou indicated the DIP payment reform achieved a short-term success in slowing down the growth of health expenditures [17].

Although the pilot programs in various locations have yielded certain results in terms of fee control and other aspects, there are also some issues that require attention and resolution in China. There remain challenges such as insufficiently refined disease classification and low motivation among medical staff. Research on the implement of SDP in a county in Shaanxi Province showed effective cost control but no significant reduction in average length of stay [18].

Despite the significance of the topic, the research content in most studies primarily focuses on specific regions or top-tier hospitals, unintentionally overlooking district hospitals, which command a substantial market share. This practice has led to research outcomes with limited generalizability due to the predominant analysis of a singular aspect. As for the research indicators, while there is ample discussion about the impact of SDP on facets like medical expenses, treatment quality, cost control, and health insurance funds, these indicators lack a systematic and comprehensive evaluation. The analysis often overlooks the internal structural changes and variable nature of medical expenses. Unfortunately, the prevailing emphasis is on the influence of SDP on medical expenses, and this evaluation often employs simplistic dimensions.

The aforementioned research questions necessitate further consideration: Firstly, this study primarily assesses the implementation impact of SDP in district hospitals, and this study addressing the dearth of relevant research on this crucial aspect of hierarchical diagnosis and treatment. Secondly, this study selects three diseases representing chronic diseases, surgical diseases, and cerebrovascular diseases representativeness. Thirdly, a multidimensional set of indicators was selected to evaluate the implementation effects of SDP. In this study, structural variation analysis and the DID model were employed to assess the impact of policy implementation. Simultaneously, through a questionnaire survey, feedback on the policy was gathered from diverse demographic groups, enhancing the clarity and alignment with regional realities regarding the reasons behind changes in medical expenses and quality.

## Methods

### Data source

The implementation of SDP was conducted in Fuzhou in August 2018, covering 141 diseases treated at district hospitals. The target population encompassed individuals covered by the city’s basic medical insurance as well as self-paying patients. The sample hospital is situated at the urban-suburban interface and serves as a district hospital. It is classified as a Grade IIA hospital within China’s hospital evaluation framework. The patient demographic predominantly consists of the elderly and migrant workers. Similar to the majority of district Grade II hospitals in China, the current state of informatics and medical records management at this district hospital remains relatively underdeveloped, with the institution still operating within the framework of SDP method. The three diseases selected for this study are Type 2 Diabetes, representing chronic conditions; Planned Caesarean Section, exemplifying surgical procedures; and Lacunar Infarction, indicative of cerebrovascular diseases common in the elderly. These were chosen based on their well-defined diagnostic criteria, established treatment protocols, and high prevalence, being among the top three conditions at the sampled hospital. Such a selection effectively mirrors the typical and recurrent diseases encountered in this hospital and, more broadly, in district healthcare settings.

**Fig. 1:**
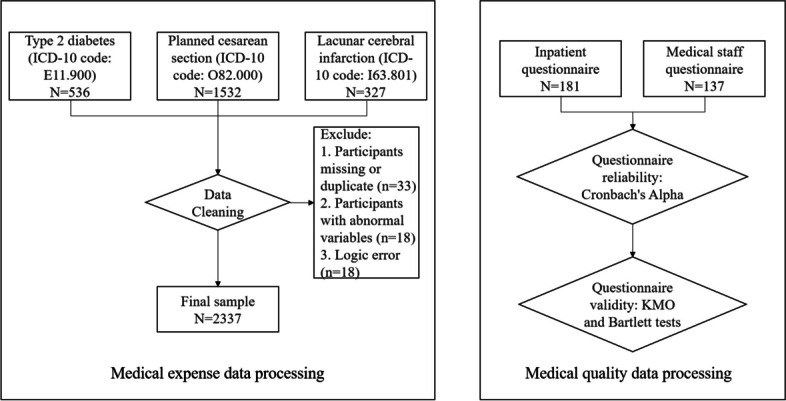
Data processing flow chart

Data was collected from the inpatient management system between January 1^st,^ 2016 and December 31st ,2021 to evaluate the impact of SDP implementation on medical expenses and patient outcomes among individuals diagnosed with these aforementioned three diseases. A total of 2395 patients with main diagnoses including type 2 diabetes, planned cesarean section and lacunar infarction were selected for inclusion in the study using medical record number for data merging purposes. After removing 58 samples according to the exclusion criteria, a final sample size of 2337 was included (See Fig. 1 for details).

The feedback on the SDP policy from inpatients and medical staff was collected through a comprehensive survey, consisting of both online and paper questionnaires. The survey for inpatients included data from 11 variables, while the survey for medical staff encompassed 22 variables. According to the sample estimation method, which suggests a minimum sample size of 5 to 10 times the number of variables, the required sample size for inpatients ranges from 55 to 110 individuals, and for medical staff, it ranges from 110 to 220 individuals. A convenience sampling method was used to select a total of 181 patients or their family members discharged between February and May 2022 from a district hospital. The surveys were administered upon their discharge, with 181 responses collected (comprising 129 electronic and 52 paper questionnaires), resulting in a response rate and effective rate of 100%. Simultaneously, surveys were distributed to 138 medical staff members (including physicians, nurses, and medical technicians) with a 100% response rate and an effective rate of 99.28%.

### Variables

After referring to existing literature [19] and thoroughly considering factors such as data accessibility, the research parameters identified in this study encompass total cost, treatment cost, drug costs, hospitalization fee, Laboratory fee, and some other fees as well as the proportion of personal payment for medical expenses. Quality of care is evaluated based on infection rate (0 = uninfected; 1 = infected), recovery rate (1 = cured; 2 = improved; 3 = uncured; 4 = other) and average length of stay.

Feedback from hospitalized patients and medical staff regarding SDP is also included in this study. The questionnaire was initially designed by reviewing and referencing relevant literature [20, 21], and further refined through expert consultation. For both inpatients and medical staff, the questionnaire consists of three parts: basic information, knowledge assessment and satisfaction assessment. The satisfaction assessment for inpatients encompasses three dimensions: medical quality, cost, and service, while for medical staffs, it includes policy quality, medical quality, new technology, doctor-patient relationship, and key performance index. A Likert 5-level scale was employed for both questionnaires. The complete questionnaire can be found in Supplementary file 1. 2.

The reliability of the questionnaire was assessed using SPSS 23.0 software. Cronbach’s Alpha values for overall satisfaction were 0.94 and 0.90, respectively, both exceeding the threshold of 0.8 (*P <* 0.05). Additionally, validity analysis was conducted using KMO and Bartlett tests which revealed KMO values of 0.904 and 0.859 for the two questionnaires (*P <* 0.05); these findings suggest satisfactory structural validity and stability. relevant data was shown in Supplementary Table 1.

### Statistical analysis

#### Descriptive analysis

The effects of SDP on medical expenses and medical quality were analyzed using the Chi-square test and one-sample t-test. The cognition level and satisfaction of SDP among hospitalized patients and medical staff and influencing factors were described by using ANOVA and Kruskal-Wallis tests. Analyses were performed using SPSS 23.0, with statistical significance defined as *P* < 0.05.

#### Structural variation analysis

The structural variation analysis method is extensively employed in the examination and investigation of medical expenses [22]. In this study, the Value of Structure Variation (VSV), Degree of Structure Variation (DSV), and Contribution of Structure Variation (CSV) are utilized to reflect the internal cost structure and dynamic changes experienced by patients in both the treatment group and control group before and after implementing SDP.

By assessing VSV, it becomes possible to gauge the direction and extent of cost changes. VSV > 0 indicates a positive change suggesting an increase in fee structure:

1 $$VSV\;=\;X_{i1}-X_{i0}$$

DSV, defined as the sum of absolute changes in each cost component during a specific period, can effectively capture the dynamic shifts in cost composition ratios over time:

2 $$DSV\;=\;\sum\left|X_{i1}\right.-X_{i0}|$$

CSV refers to the proportion of the absolute value of each cost structure change to the degree of structural change, indicates the extent to which a certain cost composition ratio affects hospitalization costs:

3 $$CSV\;=\;\left|X_{i1}-X_{i0}\right|/DSV\;\times\;100\%$$

The number zero represents the beginning of a period, while one represents the end, *X*_*i0*_ is the composition ratio of the i-th item expense at the beginning of the period.

#### Differences-in-differences (DID)

The difference-in-differences (DID) method, a widely used econometric method for evaluating the impact of policy implementation on target populations, was employed in this study to identify the effect of SDP implementation. According to the SDP policy, the specified diseases were only applicable to individuals covered by the local basic medical insurance. Those seeking medical care from outside the city were exempt from SDP policy. Consequently, the treatment group was comprised of municipal insured cases, while non-municipal insured cases constituted the control group.

4 $$Y_{it}=\alpha_0+\alpha_1Treat+\alpha_2Time+\alpha_3Treat\cdot Time+\varepsilon_{it}$$

In Eq. 4, Y_it_ is the dependent variable standing for the implement of SDP of participant i at time t. The coefficient *β*_1_ is the key parameter of interest, which captures the effect of SDP. *Treat* is a grouped dummy variable, and *Treat* = 1 if participants pertain to urban medical insurance. *Time* is a time dummy variable, and it equals one if participants collected after the implementation of SDP (2018–2021). Otherwise, it equals zero. εit is the error term. α_2_ + α_3_ represents the differential impact of SDP on *Y*_*it*_ between the treatment and control groups, capturing the pre- and post-implementation changes in *Yit* for each group separately. Specifically, α_2_ captures the within-group difference for the control group before and after SDP implementation, while α_3_ isolates the pure effect of SDP. Analyses were performed using StataSE 15 (64-bit), with statistical significance defined as *P* < 0.05.

## Results

### Sample characteristics

After data cleaning, a total of 533 cases of type 2 diabetes were identified, comprising 437 cases in the treatment group and 96 cases in the control group. No statistically significant differences were observed in terms of gender and age distribution between the two groups. Among the planned cesarean section cases (*n* = 1491), there were 349 cases in the treatment group, with no statistically significant difference observed in terms of age distribution between these two groups. Furthermore, within the subset of lacunar infarction cases (*n* = 313), there were 194 cases in the treatment group. No statistically significant disparity was found regarding age distribution between these two groups. Therefore, data collected for these three diseases are comparable in this study. Further details can be found in Supplementary Table 2.

### Hospitalization costs

#### Average cost and composition of control group and treatment group

In this study, the average hospitalization costs of the three diseases were assessed. The average hospitalization costs for three diseases exhibited a similar trend between treatment and control group. In the case of type 2 diabetes, the main contributors are drug cost, diagnostic test fee, and laboratory fees. Specifically, in 2016, drug cost accounted for 51.29% of the total cost while it constituted approximately 30% in all other years except for 2019. For patients undergoing a planned cesarean section, the main contributors are operation fees, other expenses, and drug cost. Patients with lacunar infarction tend to have higher proportions of drug cost and laboratory costs among their overall medical costs. All items show a decreasing trend after 2019, with more pronounced changes observed in the treatment group.

From 2016 to 2021, especially in 2016–2017, the structural change of the average medical cost in the treatment group was significantly greater than that in the control group (44.62%, 22.12% and 48.44%). From 2016 to 2021, the top three contributors to changes in costs for type 2 diabetes cases were drug costs, diagnostic test costs, and color ultrasound costs, accounting for a cumulative contribution rate of 78.44% in the treatment group. Notably, there was a significant decrease in drug costs. Planned cesarean section cases also experienced a significant reduction in drug costs within the treatment group from 2016 to 2017, with a contribution rate of 38.52%, while laboratory fee, nursing fee, and anesthesia fee showed positive changes. In terms of lacunar infarction cases, there was a substantial decrease of 24.22% in drug cost from 2016 to 2017 within the treatment group. However, since 2019 there has been smaller decreases or even rebounds in drug cost. Additional details can be found in Supplementary Tables 3, 4, 5, 6, 7 and 8.

#### DID analysis of medical expenses

The analysis of the DID model reveals a significant reduction in the inpatient costs for type 2 diabetes, planned cesarean section, and lacunar infarction by 28.10%, 3.30%, and 37.20% respectively (*P* < 0.05).

After implementing SDP, the treatment group showed a significant reduction in drug cost (41.50%), laboratory fee (40.80%), and color ultrasound fee (43.20%) compared to the control group (*P* < 0.05), and hospitalization fee experienced a decrease of less than 40%. As to planned cesarean section, the other expenses of the treatment group decreased by 9.20% (*P* < 0.05). Although the other items increased or decreased to different degrees, the differences were not statistically significant. The treatment group of lacunar infarction also exhibited a significant decrease in the cost of color ultrasound, bed, medicine, and nursing by 105.30%, 55.70%, 57.00%, and 55.40% respectively (*P* < 0.05), and a significant increase in operation fee by 40.60% (*P* < 0.05). Other changes are presented in Table 1.

**Table 1:** The change of hospitalization expenses among treatment and control groups (China, 2016–2021)

Expense categories(yuan)	Treatment group		Control group		DIFF	S_DIFF_	* P*-value
	pre-implementation	post-implementation	pre-implementation	post-implementation			
**panel A: type 2 diabetes**							
Total cost	6774.70	7053.85	7500.69	5609.44	-0.281	0.069	0.000***
Treatment cost	236.16	315.90	254.19	272.30	-0.099	0.153	0.515
Drug cost	2380.28	2385.71	2727.16	1763.17	-0.415	0.160	0.010***
Hospitalization fee	322.26	334.31	434.32	291.70	-0.358	0.124	0.004***
Laboratory fee	125.74	136.80	151.55	121.86	-0.266	0.109	0.015**
Check-up fee	533.23	906.83	601.93	791.67	-0.205	0.164	0.212
Operation fee	0.00	51.58	1.99	104.76	0.630	0.643	0.333
Diagnostic test fee	1826.32	1653.67	1831.47	1150.24	-0.408	0.104	0.000***
CT fee	521.42	383.45	561.20	359.64	-0.197	0.117	0.095
Color ultrasound fee	374.48	308.89	418.02	233.47	-0.432	0.181	0.018**
Nursing fee	58.42	60.86	94.56	100.33	2.410	6.146	0.695
Anesthetic fee	243.00	254.49	279.80	217.49	-0.255	0.110	0.021**
Other expenses	153.39	261.36	144.52	202.81	-0.150	0.174	0.390
**panel B: planned cesarean section**							
Total cost	7222.25	7142.75	7085.49	6773.52	-0.033	0.013	0.008***
Treatment cost	313.23	337.04	307.91	313.02	-0.050	0.030	0.105
Drug cost	1345.88	1257.96	1242.62	1179.87	0.009	0.030	0.757
Hospitalization fee	747.57	686.25	779.65	630.81	-0.137	0.072	0.057
Laboratory Fee	289.23	548.67	297.83	532.15	-0.050	0.277	0.213
Check-up fee	89.77	96.25	87.84	90.95	-0.034	0.044	0.260
Operation Fee	1433.76	1438.73	1427.77	1413.00	-0.010	0.017	0.567
Diagnostic test fee	459.53	480.71	432.69	470.12	0.069	0.041	0.093
Color ultrasound fee	42.77	53.32	41.98	32.52	-0.068	0.569	0.233
Nursing fee	568.82	603.80	565.11	601.94	-0.019	0.198	0.344
Anesthetic fee	370.82	421.67	366.18	411.61	-0.015	0.280	0.592
Other expenses	1560.89	1218.34	1535.93	1097.54	-0.092	0.029	0.001***
**panel C: Lacunar infarction**							
Total cost	8083.86	7806.41	8834.50	5432.32	-0.372	0.101	0.000***
Treatment cost	411.23	399.75	354.50	283.58	-0.269	0.163	0.100
Drug costs	3937.54	3381.31	3949.50	1847.46	-0.557	0.153	0.000***
Hospitalization fee	383.50	406.10	508.39	277.00	-0.570	0.133	0.000***
Laboratory Fee	568.70	765.32	719.73	651.29	-0.380	0.189	0.045**
Check-up fee	147.75	149.63	182.54	113.72	-0.472	0.118	0.000***
Operation Fee	0.00	5.76	0.00	120.35	0.406	0.069	0.000***
Diagnostic test fee	1315.32	1153.94	1497.53	923.70	-0.299	0.135	0.028**
CT fee	315.42	345.08	541.85	337.36	-0.389	0.176	0.029**
Color ultrasound fee	166.57	44.98	297.96	204.19	1.053	0.255	0.000***
Nursing fee	6.27	0.85	11.54	7.85	-1.276	1.152	0.280
Anesthetic fee	376.17	417.81	361.26	202.13	-0.554	0.151	0.000***
Other expenses	455.41	735.87	409.68	463.72	-0.509	0.232	0.029**

Significance levels *P < 0.1; **P < 0.05; ***P < 0.01

#### The influence of SDP on individual payment ratio

Table 2 reports the influence of SDP on the proportion of individual payment for patients with three diseases was analyzed using a single-sample t-test. All data passed normality and homogeneity of variance tests.

**Table 2:** The influence of SDP on the proportion of individual payment ratio

Medical insurance type	Personal payment ratio (%)		t-value	Prob
	pre-implementation	post-implementation		
**panel A: type 2 diabetes**				
working staff	26.60	20.00	2.804	0.014***
retired staff	23.89	15.00	15.467	0.000***
urban and rural residents	34.81	35.00	-2.184	0.051
**panel B: planned cesarean section**				
working staff	31.88	20.00	17.519	0.000***
urban and rural residents	50.37	35.00	6.523	0.000***
**panel C: Lacunar infarction**				
working staff	28.90	20.00	10.787	0.000***
retired staff	21.85	15.00	5.438	0.000***
urban and rural residents	31.26	35.00	-5.740	0.000***

Significance levels *P < 0.1; **P < 0.05; ***P < 0.01

The individual payment ratios for on-the-job employees, retired employees, and urban and rural residents participating in insurance are 20%, 15%, and 35% respectively. Following the implementation of SDP, there was a significant decrease in the proportion of individual payment for type 2 diabetes and lacunar infarction among both active and retired medical insurance workers (*P <* 0.05), indicating a statistically significant difference. In contrast, for urban and rural residents with medical insurance, there was a significant increase in the proportion of individual payment for lacunar infarction before and after the implementation of SDP (*t* = -5.74, *P <* 0.05).

Furthermore, planned cesarean section resulted in a significant decrease in health care coverage for employed individuals as well as a decrease in the individual payment ratio for urban and rural residents’ health care (*P <* 0.05), demonstrating statistical significance.

### Medical quality

In this study, the average duration of hospitalization and cure rate were utilized as indicators to assess the changes in medical quality for a specific disease before and after the implementation of SDP. As planned cesarean section is an invasive procedure, the infection rate associated with was used as an evaluation indicator. The DID model analysis revealed that prior to, during, and after the implementation of type 2 diabetes group hospitalization days decreased by 2.438 days (*P <* 0.05). Furthermore, there was a statistically significant decrease in the infection rate of planned cesarean section group by 0.012% (*P <* 0.05). Additionally, in the treatment group, there was a statistically significant increase in the cure rate of lacunar infarction by 8.99%, accompanied by a reduction in hospital stay duration by 4.268 days (*P <* 0.05). Further details were shown in Table 3.

**Table 3:** The change of medical quality among treatment and control groups (China, 2016–2021)

Expense categories(yuan)	Treatment group		Control group		DIFF	S_DIFF_	* P*-value
	pre-implementation	post-implementation	pre-implementation	post-implementation			
**panel A: type 2 diabetes**							
Cure rate (%)	87.10	90.77	91.81	96.53	1.060	4.905	0.834
Average length of stay (days)	7.71	7.60	9.22	6.66	-2.438	0.700	0.001***
**panel B: planned cesarean section**							
Cure rate (%)	99.37	94.80	99.54	95.49	2.127	3.765	0.588
Infection rate (%)	1.13	0.87	1.39	0.00	-0.012	0.003	0.006***
Average length of stay (days)	5.67	5.43	5.73	5.25	-0.242	0.263	0.357
**panel C: Lacunar infarction**							
Cure rate (%)	86.67	86.44	89.91	97.65	8.990	5.393	0.049**
Average length of stay (days)	8.87	8.46	10.96	6.11	-4.268	0.894	0.000***

Significance levels *P < 0.1; **P < 0.05; ***P < 0.01

### Parallel trend test and placebo test

**Fig. 2:**
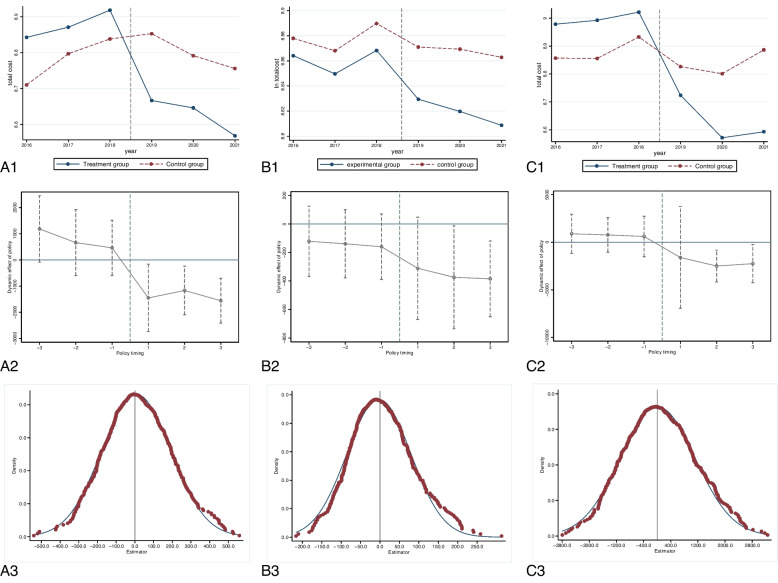
Illustrates the time trend chart, dynamic effect test chart and Placebo test chart of hospitalization expenses for both the experimental group and the control group across three diseases Notes: picture A represents Type 2 diabetes, picture B represents Planned cesarean section, and picture C represents lacunar infarction; picture 1 is Time trend Chart, picture 2 is Dynamic effect test chart, while picture 3 is Placebo chart

The parallel trend tests are conducted to ensure that both the control group and the treatment group exhibit similar trends prior to policy implementation when utilizing the DID model for evaluating the impact of SDP. Figure 2 illustrates that before and after the policy implementation (August, 2018), there was a comparable change trend in hospitalization costs between the treatment and control groups across three diseases, essentially met the parallel trend test. Furthermore, dynamic effects were examined between these two groups across three diseases. Prior to SDP implementation (before1, before2 and before3), there was no significant difference between the control group and the treatment group before the implementation of the policy. Following SDP adoption (after1, after2 and after3), however, only during the first year of implementation did confidence intervals exclude zero; signifying a significant disparity between control and treatment groups post-policy enactment with a slight delay in its impact. Consequently, the DID model adheres to parallel trend testing standards while yielding more robust and effective results.

### State feedback policy

The average satisfaction of inpatients receiving SDP was 4.46 points, indicating high overall satisfaction with this payment model. After analyzing various dimensions of satisfaction, it was found that medical expenses received the highest score (4.56 points), followed by satisfaction with medical quality and service. Medical quality satisfaction encompasses treatment effectiveness, professional competence, technical proficiency, and length of hospital stay. Among these factors, respondents expressed the highest level of satisfaction with the length of stay. Supplementary Table 9 provides more detailed information.

The level of understanding and the composition of understanding methods among the sample inpatients regarding SDP were analyzed. A significant proportion (46.96%) of patients exhibited a high level of familiarity with SDP, and the primary sources through which most patients acquired knowledge about SDP were medical staff and hospital publicity, accounting for 90.60%, whereas only 5.50% of patients reported not having heard about it (See in Supplementary Table 10).

The correlation between patients’ department and medical insurance category and their overall satisfaction with SDP was analyzed using ANOVA. Additionally, the relationship between patients’ understanding of SDP and their overall satisfaction was examined using Kruskal Wallis Test. As shown in Table 4, there were significant differences in overall satisfaction levels among patients from different departments (χ2 = 2.763, *P* < 0.05). Obstetrics and gynecology had the highest average overall satisfaction score of 4.73 points, while pediatrics had the lowest average score of 4.04 points.

**Table 4:** Correlation analysis of payment satisfaction of hospital patients with SDP

variables	N	mean value	SD	χ2	Prob
Departments					
Internal medicine	57.00	4.35	0.760	2.763	0.030**
Surgery department	27.00	4.48	0.620		
Gynecology and obstetrics	21.00	4.73	0.490		
pediatric department	13.00	4.04	0.730		
emergency department	44.00	4.57	0.640		
Medical insurance category					
Fujian medical insurance	18.00	4.74	0.440	1.711	0. 167
Fuzhou medical insurance	121.00	4.45	0.690		
Remote medical insurance	14.00	4.27	0.760		
No medical insurance	9.00	4.25	0.83		
knowledge levels					
Not knowledgeable at all	2.00	3.21	0.510	54.793	0.000***
Slightly knowledgeable	18.00	3.77	0.950		
Moderately knowledgeable	24.00	3.85	0.730		
Very knowledgeable	52.00	4.37	0.530		
Extremely knowledgeable	85.00	4.82	0.370		

Significance levels *P < 0.1; **P < 0.05; ***P < 0.01

Supplementary Table 10 shows that the medical staff in this hospital exhibited an average overall satisfaction score of 3.86 points for SDP, indicating a level above the median. Their highest satisfaction was observed in the doctor-patient relationship (4.08 points). Following closely is their satisfaction with SDP (4.07 points). Efficiency and income-related contentment are influenced by three sub-indices: income levels, incentive mechanisms workload efficiency; notably though their lowest score was recorded for incentive mechanisms (3.72 points).

The assessment of the level of comprehension regarding SDP among medical personnel and its implementation strategies revealed that 27.74% of exhibited limited or no understanding of this policy. A majority (95.60%) reported awareness through hospital publicity, while a minority (1.50%) indicated unfamiliarity with its existence (See in Supplementary Table 12).

The data presented in Table 5 demonstrates a significant association between the age, job position, educational background of medical personnel and their overall satisfaction with payment for a specific illness. Medical staff’s overall satisfaction varied based on different age groups in response to macro-reforms, with those aged 50–60 exhibiting the lowest level of satisfaction (3.47 points) while those aged 20–30 had the highest level (4.02 points). Overall satisfaction demonstrated a declining trend as age increased, which was statistically significant (*χ2* = 2.738, *P <* 0.05).

There were differences in overall satisfaction among medical staff in different positions; doctors had the lowest average score (3.72 points), while nursing staff and medical technicians scored an average of 4.02 and 3.75 points respectively, which showed statistical significance (χ2 = 3.314, *P* < 0.05). Medical staff also exhibited significant differences in overall satisfaction based on their educational background (χ2 = 3.279, *P* < 0.05). Supplementary Table 13 indicated that insufficient disease coverage is widely recognized as the main challenge in implementing SDP at this stage (72.30%). Additionally, limited patient awareness and acceptance rate (67.20%) also hinder its effectiveness.

**Table 5:** Correlation analysis of payment satisfaction of medical staffs with SDP

variables	N	mean value	SD	χ2	Prob
Age					
20-30years	43.00	4.02	0.720	2.738	0.046**
30-40years	53.00	3.91	0.68		
40-50years	30.00	3.70	0.610		
50-60years	11.00	3.47	0.43		
Job title					
Primary title	71.00	3.96	0.650	7.840	0.020**
Middle title	47.00	3.85	0.74		
High title	19.00	3.54	0.510		
Job positions					
Physician	54.00	3.72	0.610	3.314	0.039**
Nurse	63.00	4.02	0.72		
Medical technician	20.00	3.75	0.630		
Moderately knowledgeable					
College or below	40.00	4.08	0.71	3.279	0.041**
Undergraduate	92.00	3.76	0.63		
Master degree or above	5.00	4.01	0.92		
knowledge levels					
Not knowledgeable at all	1.00	4.47	-	26.215	0.000***
Slightly knowledgeable	3.00	3.33	0.53		
Moderately knowledgeable	34.00	3.52	0.480		
Very knowledgeable	47.00	3.71	0.54		
Extremely knowledgeable	52.00	4.24	0.72		

Significance levels *P < 0.1; **P < 0.05; ***P < 0.01

## Discussion

### The implementation of SDP has a significant impact on medical expenses

Compared to the control group, the implementation of SDP resulted in varying degrees of reduction in both medical costs and individual payment ratios. Notably, in comparison with planned cesarean sections, the drug costs for the other two diseases decreased most significantly. Building on previous research on cost control [23], it is evident that SDP has a substantial impact on cost containment. This phenomenon can be attributed to two underlying factors: firstly, the SDP policy establishes payment limits based on disease classification, with any excess covered by the hospital. In an effort to curb excessive out-of-pocket medical expenses, hospitals actively reduce treatment costs and avoid unnecessary diagnostic procedures, consequently leading to decreased hospitalization costs for patients. Secondly, medical insurance agencies establish completion indicators for SDP, which are included in hospital performance evaluations. This prompts hospital supervision departments to monitor the implementation of SDP more closely. The pressure exerted by performance evaluations drives an annual increase in the completion rate. Moreover, clinical departments proactively control costs and meet target requirements by obtaining surplus funds while simultaneously alleviating financial burdens on patients. Therefore, SDP indeed plays a positive role in controlling medical expenses.

### SDP can optimize the medical expenses structure to some extent

#### The proportion of drug expenses decreased, and the proportion of inspection and inspection expenses increased

In April 2017, the National Health Commission of the People’s Republic of China and seven other departments jointly issued a notice proposing the commission of drugs in public hospitals, aiming to reduce the proportion of drug costs by 30% at the end of 2017. Concurrently, Fuzhou implemented SDP that year. The groups with type 2 diabetes and lacunar infarction witnessed respective decreases in drug costs. Even in cases of planned cesarean section, where treatment costs typically predominate, both groups observed a proportional decline in drug costs. Thus, SDP demonstrated its efficacy in mitigating the proportion of drug costs.

It is worth noting that the proportion of drug costs significantly declined in 2016–2017, while inspection costs such as laboratory fees and CT fees increased. Medical staff may compensate for this loss and generate additional revenue by prescribing more diagnostic procedures. Additionally, similar to the investigation conducted by X. Zhang et al. [24], which demonstrates that controlling the proportion of drugs will induce an increase in examination demand, resulting in excessive medical treatment. It is also possible that doctors, in order to meet the required target of drug cost ratios, increased the proportion of laboratory fees [25]. Supervision over drug proportions experienced a slowdown after 2019, resulting in varying degrees of rebound in drug costs for these three diseases. Consequently, doctors adopted a passive approach towards examinations without fully recognizing the significance of rational drug use and cost control. The management of drug costs remains a focal point in ongoing healthcare reform endeavors.

#### The proportion of medical service expenses is relatively low

Medical service fees, encompassing examinations, nursing care, treatments, and operations, more accurately reflect the labor value and technological complexity involved. Research indicates that the current pricing of medical service fees in public hospitals significantly underestimates the labor value of healthcare professionals [26]. In recent years, increased monitoring measures have led to higher costs associated with these services. This study observed a notable rise in medical service expenses within the treatment group from 2016 to 2021, suggesting that the Service Delivery Policy (SDP) has moderately increased funding allocation for medical services.

Aligned with previous findings indicating a low proportion of medical service costs and an unbalanced cost structure [25], this study noted that, with the exception of the medical service cost for planned cesarean sections, the overall increase in the medical service cost for other diseases was modest and constituted a relatively small proportion of total expenses.

#### The proportion of other expenses, mainly consumables, decreased

In the detailed breakdown of hospitalization expenses at this sample hospital, single-use consumables, consultation fees, and miscellaneous costs are collectively categorized under ‘other expenses’. The proportion of these miscellaneous costs in cases of planned cesarean sections was notably higher than in other diseases from 2016 to 2021, ranking second only to surgical and drug cost. This increase is attributed to the necessities of analgesia and postoperative care in surgical conditions.

During the same period, the ‘other expenses’ for planned cesarean sections showed a declining trend. A DID analysis of medical expenses revealed that the ‘other expenses’ for planned cesarean sections in the treatment group decreased significantly compared to the control group. Consequently, our findings closely align with those of Zhang Tao [27], suggesting that the implementation of the SDP can play a significant role in controlling the proportion of consumable costs.

### The implementation of SDP can effectively improve the quality of medical treatment

In this study, quality of medical care was evaluated based on cure rate, infection rate, and average length of stay. After the implementation of the SDP, we observed significant differences in these indicators between the treatment group and the control group.

The SDP potentially enhances medical quality indicators via two key strategies: First, integrating the SDP with clinical pathways reduces variability in treatment plans and standardizes diagnostic procedures, and avoids unnecessary hospital stays, thereby improving medical resource utilization [28]. Secondly, the SDP compels healthcare institutions to achieve profitability by providing patient diagnosis and treatment within preset budgets. This incentivizes hospitals to standardize their operations, ensuring safer, more effective, and cost-efficient medical services for patients. Consequently, both the quality and safety of medical care for specific diseases have significantly improved.

### Patients’ understanding of SDP needs to be strengthened

In this study, patients’ satisfaction with SDP was significantly high. The greatest satisfaction was observed in terms of medical expenses, particularly the reimbursement ratio for hospitalization costs and the average length of stay. In contrast, the service attitude of medical staff received relatively lower satisfaction ratings.

The implementation of the SDP significantly reduced the individual payment burden for patients compared to periods without SDP, effectively decreasing overall medical costs, hospitalization expenses, costs of accompanying care, and average length of stay, thereby alleviating the disease burden on patients. As highlighted by Lu Peiy et al. [29], these factors significantly contribute to enhanced patient satisfaction post-SDP implementation.

The primary method through which patients learn about the SDP is via medical and hospital publicity; however, about 24.30% of patients still possess a limited understanding of the SDP. Patient satisfaction tends to increase with improved information symmetry between patients and medical staff regarding the SDP. Therefore, a lack of sufficient patience from medical staff in informing patients about the SDP could lead to diminished satisfaction with their service attitude.

### The efficiency of SDP executed by medical staffs is low

In this study, the overall satisfaction of medical staff with the SDP was above average. Notably, the highest satisfaction pertained to the doctor-patient relationship, whereas satisfaction with new technologies and projects was the lowest.

The implementation of the SDP has significantly influenced cost control and quality improvement, thus fostering more harmonious doctor-patient relationships. It has also contributed to a reduction in the average length of stay and improved medical resource utilization, leading to increased satisfaction among medical staff in terms of workload efficiency and hospital stay durations [30].

The introduction of new technologies and projects within the scope of this study was associated with increased medical costs. Some researchers propose that pre-payment methods like DRGs might encourage the adoption of new technologies [31]. Yet, in China, the execution of the SDP has curtailed the usage of new technologies and diagnostic methods due to cost control measures and the status quo of public hospitals. Post-SDP implementation, physicians are required to adjust their diagnosis and treatment plans to comply with established protocols, taking into account the individual needs of patients. Consequently, this has led to lower satisfaction levels concerning new technologies and changes in treatment practices.

The study also revealed that satisfaction with the SDP varied among medical staff based on professional title and understanding of the SDP. Higher professional titles correlated with lower satisfaction levels, whereas a better understanding of the SDP was associated with higher satisfaction. Furthermore, medical personnel identified persistent issues in the implementation of the SDP, such as restricted coverage for conditions with clear diagnoses and simplicity, and inflexible pricing, which becomes problematic when personalized treatment methods or new technological projects are applied [32].

### Suggestions for the implementation SDP

Based on the results of policy interventions and considering the development level of district hospitals and the characteristics of mass cognition, this study proposes several strategies to optimize the SDP.

1. For the district hospitals with a very weak reform basis such as medical record data, information conditions, and talent teams, the key to connecting DRG or DIP payment methods is to expand the coverage of diseases and improve the policy system such as SDP negotiation procedures, pricing standards, and incentive mechanisms. To achieve this objective, district hospitals can focus on two areas of change: (1) The district hospitals may request the government to enhance financial subsidies to fortify their own information infrastructure, such as electronic health records (EHR) [33], thus reduce reliance on third-party software providers. (2) The quality of medical records is the main obstacle to enrollment. It is crucial to enhance focus on medical record management and coding tasks in district hospitals, which will expedite reforms in methods for medical insurance payment.
2. Enhance publicity and management system. Relevant institutions should prioritize addressing patients’ concerns about SDP in order to facilitate effective doctor-patient communication and public oversight. Some senior medical staff with prestigious professional titles may have misconceptions about SDP, thus it is imperative to reinforce their scientific understanding of SDP starting from the leadership level. Hospitals should actively monitor the implementation of SDP and allocate resources based on departmental characteristics to achieve desired outcomes. Additionally, a well-defined reward and punishment system should be established to enhance the motivation of medical personnel.
3. Improve policies and optimize cost structure. The medical insurance department should expand disease coverage and refine the entry-exit mechanism. Additionally, improving centralized drug procurement policies and sharing the medical examination information to reduce drug costs and laboratory fee. It is also necessary to adjust medical service prices reasonably based on regional disparities and hospital grades, gradually increase charges for medical services, encourage doctors’ proactive engagement in health education and disease management activities, promote hierarchical diagnosis and treatment approaches, and facilitate integration between healthcare provision and prevention efforts.

## Conclusions

This study integrated literature analysis and field investigation, employing structural variability and differential models to effectively address the endogeneity issue, accurately evaluate the implementation impact of SDP policy, enhance the application of structural variability analysis and differential models in the medical and health domain, leading to the following conclusions: (1) The implementation of SDP significantly impacts medical expenses, leading to varying reductions in hospitalization costs for the three diseases. Additionally, SDP optimizes cost structure by significantly decreasing the proportion of drug and consumables expenses. However, there are still concerns regarding the low proportion of medical service costs and an increase in laboratory and check-up fees. (2) The implementation of SDP effectively enhances healthcare quality by improving recovery rates, reducing infection rates, and decreasing average length of stay. Subjective quality assessments indicate high patient satisfaction with SDP; but their understanding and comprehension of it need reinforcement. In contrast, the medical staff’s satisfaction level regarding SDP exceeds the average; they find the implementation challenging and lack enthusiasm for active participation. (3) The implementation of SDP may give rise to certain undesirable phenomena that can be mitigated by improving information technology levels and medical record management in district hospitals, along with expediting the progress of reforms in medical insurance payment methods.

Due to data acquisition challenges and limited research capacity, the study has these limitations: (1) Some crucial indicators may have been overlooked due to constraints imposed by hospital informatization levels and medical record writing quality leading to restricted results; (2) The satisfaction survey of SDP was conducted solely post-implementation without examining the difference in satisfaction before and after its implementation; (3) This study was limited to analyzing data from only one hospital and three diseases that met the research criteria and were representative of the policy impact.

Further research is necessary to address these limitations. It should include other district hospitals to ensure objective data collection; incorporate a broader range of diseases that offer significant representativeness; refine the indicators for assessing the impact of medical insurance payment reform; and conduct comparative analyses of satisfaction surveys conducted before and after the reform. These efforts will provide scientific references for district hospitals to improve their SDP systems and gradually transition scientific and refined payment methods.

### Supplementary Information

**Supplementary Material 1.**

## Data Availability

The datasets used and analysed during the current study are available from the corresponding author on reasonable request. Supplementary information files during this study are included in this published article.

## Abbreviations

SDP: Single Disease Payment
DID: Differences-in-Differences
DRGs: Disease diagnosis group system
DRG-PPS: A pre-payment system based on disease diagnosis group
DIP: Diagnosis Intervention Packet
EHR: Electronic health records
