# Supplementary material for: Can single disease payment impact hospitalization expenses and quality in district hospital? A case study in Fujian, China
Source: Int J Equity Health. 2024 Mar 13;23:53. doi: 10.1186/s12939-024-02134-2 (PMC10938684; doi:10.1186/s12939-024-02134-2)
Supplement: Supplementary file 1 — Supplementary Material 1. [file 12939_2024_2134_MOESM1_ESM.docx]

**Supplementary material**

**Content**

[Supplementary results 2](#_Toc147528333)

[Supplementary Table 1 Satisfaction questionnaire reliability test table 2](#_Toc147528334)

[Supplementary Table 2 Distribution of case variables in different disease groups and control groups. 2](#_Toc147528335)

[Supplementary Table 3 Average hospitalization cost of Type 2 diabetes mellitus (Yuan) and component ratio (%) 3](#_Toc147528336)

[Supplementary Table 4 Average hospitalization cost of planned cesarean section mellitus (Yuan) and component ratio (%) 4](#_Toc147528337)

[Supplementary Table 5 Average hospitalization cost of lacunar cerebral infarction mellitus (Yuan) and component ratio (%) 5](#_Toc147528338)

[Supplementary Table 6 The change value, change degree and contribution rate of type 2 diabetes inpatient cost structure 6](#_Toc147528339)

[Supplementary Table 7 The change value, change degree and contribution rate of planned cesarean section inpatient cost structure 7](#_Toc147528340)

[Supplementary Table 8 The change value, change degree and contribution rate of lacunar cerebral infarction inpatient cost structure 8](#_Toc147528341)

[Supplementary Table 9 Inpatients' satisfaction with dimensions and each dimension of secondary indexes 9](#_Toc147528342)

[Supplementary Table 10 Analysis of in-patient awareness of payment for single disease 9](#_Toc147528343)

[Supplementary Table 11 Medical staffs' satisfaction with dimensions and each dimension of secondary indexes 10](#_Toc147528344)

[Supplementary Table 12 Analysis of medical staff awareness of payment for single disease 11](#_Toc147528345)

[Supplementary Table 13 Problems in the implementation of SDP 11](#_Toc147528346)

[Questionnaire survey 12](#_Toc147528347)

[Supplementary File 1 The inpatients’ satisfaction on the implement of SDP Questionnaire (in the original languages and translated) 12](#_Toc147528348)

[Supplementary File 2 The medical staffs’ satisfaction on the implement of SDP Questionnaire (in the original languages and translated) 14](#_Toc147528349)

# Supplementary results

Supplementary Table 1 Satisfaction questionnaire reliability test table

| **Dimension** |  | Cronbach’s Alpha | Terms number |
| --- | --- | --- | --- |
|  |  |  |  |
| Inpatient |  |  |  |
| Medical quality |  | 0.897 | 3 |
| Medical costs |  | 0.893 | 2 |
| Medical service |  | 0.794 | 2 |
| Medical staff |  |  |  |
| Quality of policy |  | 0.914 | 3 |
| Medical costs |  | 0.752 | 5 |
| New technology/projects |  | 0.973 | 2 |
| New technology new projects |  | 0.707 | 3 |
| physician-patient relationship |  | 0.950 | 2 |

Supplementary Table 2 Distribution of case variables in different disease groups and control groups.

| **variables** | Treatment | Control | *χ2* | *P*-value |
| --- | --- | --- | --- | --- |
| **panel A：type 2 diabetes** | |  |  |  |
| Gender |  |  |  |  |
| male | 207(47.37) | 54(56.25) | 2.485 | 0.115 |
| female | 230(52.63) | 42(43.75) |  |  |
| Age |  |  |  |  |
| ＜40 years | 21(4.81) | 7(7.23) | 3.720 | 0.293 |
| 41-60 years | 138(31.58) | 37(38.54) |  |  |
| 61-80 years | 247(56.52) | 48(50.00) |  |  |
| ＞80 years | 31(7.10) | 4(4.12) |  |  |
| **panel B：planned cesarean section** | | |  |  |
| Age |  |  |  |  |
| ＜28 years | 117(40.28) | 460(33.52) | 8.817 | 0.066 |
| 29-32 years | 139(33.19) | 379(39.83) |  |  |
| 33-35 years | 52(13.75) | 157(14.90) |  |  |
| 36-39 years | 30(8.46) | 97(8.60) |  |  |
| ＞40 years | 11(4.29) | 49(3.15) |  |  |
| **panel C：Lacunar infarction** | |  |  |  |
| Gender |  |  |  |  |
| male | 106(54.64) | 74(62.18) | 1.719 | 0.190 |
| female | 88(45.36) | 45(37.82) |  |  |

Supplementary Table 3 Average hospitalization cost of Type 2 diabetes mellitus (Yuan) and component ratio (%)

| **Expense categories** | 2016 | |  | 2017 | |  | 2018 | |  | 2019 | |  | 2020 | |  | 2021 | |
| --- | --- | --- | --- | --- | --- | --- | --- | --- | --- | --- | --- | --- | --- | --- | --- | --- | --- |
|  | Treatment (n = 85) | Control  (n =9) |  | Treatment (n = 89) | Control  (n = 9) |  | Treatment (n = 119) | Control  (n = 13) |  | Treatment (n = 13) | Control  (n = 20) |  | Treatment (n = 72) | Control  (n = 19) |  | Treatment (n =59) | Control  (n = 26) |
| Treatment cost | 226.06 (3.66) | 254.77 (3.46) |  | 302.43 (4.46) | 262.40 (3.53) |  | 197.27 (2.74) | 247.62 (3.24) |  | 232.27 (3.15) | 151.19 (2.55) |  | 373.21 (5.35) | 243.70 (4.20) |  | 338.35 (4.93) | 333.88 (6.29) |
| Drug costs | 3151.37 (51.04) | 3773.67 (51.29) |  | 2018.98 (29.79) | 2367.17 (31.83) |  | 2096.58 (29.17) | 2248.88 (29.40) |  | 1889.16 (25.59) | 1042.47 (17.59) |  | 2630.84 (37.75) | 1992.76 (34.35) |  | 2588.53 (37.72) | 1641.80 (30.95) |
| Hospitalization fee | 311.11 (5.04) | 492.18 (6.69) |  | 302.78 (4.47) | 451.0 (6.06) |  | 343.46 (4.78) | 380.5 (4.97) |  | 328.25 (4.45) | 251.9 (4.25) |  | 360.00 (5.17) | 332.78 (5.74) |  | 320.19 (4.67) | 250.34 (4.72) |
| Laboratory Fee | 219.33 (3.55) | 300.92 (4.09) |  | 544.33 (8.03) | 619.66 (8.33) |  | 742.85  (10.33) | 803.66 (10.51) |  | 1052.55 (14.26) | 996.62 (16.81) |  | 835.05 (11.98) | 795.78 (13.72) |  | 847.19 (12.34) | 741.49 (13.98) |
| Check-up fee | 101.11 (1.64) | 134.65 (1.83) |  | 130.00 (1.92) | 175.15 (2.35) |  | 139.85 (1.95) | 145.97 (1.91) |  | 140.40 (1.90) | 120.46 (2.03) |  | 142.11 (2.04) | 130.11 (2.24) |  | 130.15 (1.90) | 112.10 (2.11) |
| Operation Fee | 0.00 (0.00) | 5.29 (0.07) |  | 0.00 (0.00) | 1.48 (0.02) |  | 0.00 (0.00) | 0.00 (0.00) |  | 35.38 (0.48) | 54.61 (0.92) |  | 70.26 (1.01) | 59.10 (1.02) |  | 50.39 (0.73) | 171.53 (3.23) |
| Diagnostic test fee | 1368.67 (22.17) | 1322.11 (17.97) |  | 1980.44 (29.22) | 1988.62 (26.74) |  | 2036.46 (28.33) | 2077.78 (27.16) |  | 2129.40 (28.85) | 1922.83 (32.44) |  | 1364.32 (19.57) | 1145.10 (19.74) |  | 1499.16 (21.84) | 986.29 (18.59) |
| CT fee | 191.33 (3.10) | 360.78 (4.90) |  | 605.89 (8.94) | 565.29 (7.60) |  | 691.46 (9.62) | 701.29 (9.17) |  | 562.80 (7.62) | 494.77 (8.35) |  | 268.00 (3.85) | 329.86 (5.69) |  | 329.85 (4.81) | 366.20 (6.90) |
| Color ultrasound fee | 291.89 (4.73) | 271.22 (3.69) |  | 302.44 (4.46) | 382.12 (5.14) |  | 481.54 (6.70) | 549.73 (7.19) |  | 552.40 (7.48) | 583.62 (9.85) |  | 272.11 (3.90) | 188.68 (3.25) |  | 148.46 (2.16) | 210.97 (3.98) |
| Nursing fee | 180.44 (2.92) | 229.94 (3.13) |  | 260.00 (3.84) | 332.07 (4.46) |  | 274.54 (3.82) | 276.31 (3.61) |  | 275.40 (3.73) | 214.39 (3.62) |  | 251.84 (3.61) | 238.47 (4.11) |  | 240.35 (3.50) | 192.56 (3.63) |
| Anesthetic fee | 3.33 (0.05) | 74.15 (1.01) |  | 123.44 (1.82) | 138.84 (1.87) |  | 51.54 (0.72) | 76.03 (0.99) |  | 12.40 (0.17) | 4.46 (0.08) |  | 106.42 (1.53) | 121.69 (2.10) |  | 64.85 (0.94) | 95.39 (1.80) |
| Other expenses | 129.81 (2.10) | 138.05 (1.88) |  | 207.48 (3.06) | 153.88 (2.07) |  | 132.26 (1.84) | 142.15 (1.86) |  | 170.92 (2.32) | 90.47 (1.53) |  | 295.63 (4.24) | 223.39 (3.85) |  | 305.89 (4.46) | 202.46 (3.82) |
| All-in cost | 6174.46 (100.00) | 7357.72 (100.00) |  | 6778.23 (100.00) | 7437.70 (100.00) |  | 7187.81 (100.00) | 7649.92 (100.00) |  | 7381.34 (100.00) | 5927.80 (100.00) |  | 6969.78 (100.00) | 5801.42 (100.00) |  | 6863.36 (100.00) | 5305.00 (100.00) |

Supplementary Table 4 Average hospitalization cost of planned cesarean section mellitus (Yuan) and component ratio (%)

| **Expense categories** | 2016 | |  | | 2017 | |  | 2018 | |  | 2019 | |  | 2020 | |  | 2021 | |
| --- | --- | --- | --- | --- | --- | --- | --- | --- | --- | --- | --- | --- | --- | --- | --- | --- | --- | --- |
|  | Treatment (n = 62) | Control (n = 330) |  | Treatment (n = 70) | | Control (n = 260) |  | Treatment (n = 84) | Control (n = 206) |  | Treatment (n = 30) | Control (n = 136) |  | Treatment (n = 36) | Control (n = 74) |  | Treatment (n = 67) | Control (n = 136) |
| Treatment cost | 302.67 (4.25) | 302.26 (4.17) |  | 306.02 (4.37) | | 315.68 (4.41) |  | 313.34 (4.40) | 327.70 (4.50) |  | 306.60 (4.48) | 339.31 (4.74) |  | 290.38 (4.28) | 334.52 (4.67) |  | 328.06 (4.87) | 336.14 (4.72) |
| Drug costs | 1460.64 (20.51) | 1540.54 (21.27) |  | 1104.47 (15.76) | | 1168.84 (16.35) |  | 1196.81 (16.80) | 1257.50 (17.27) |  | 1221.56 (17.84) | 1250.59 (17.47) |  | 1186.65 (17.49) | 1246.48 (17.41) |  | 1157.57 (17.19) | 1271.58 (17.86) |
| Hospitalization fee | 735.12 (10.32) | 710.60 (9.81) |  | 867.21 (12.37) | | 774.02 (10.83) |  | 739.55 (10.38) | 773.40 (10.62) |  | 612.92 (8.95) | 727.22 (10.16) |  | 536.11 (7.90) | 611.76 (8.55) |  | 689.70 (10.24) | 685.81 (9.63) |
| Laboratory Fee | 209.37 (2.94) | 216.89 (3.00) |  | 256.90 (3.67) | | 284.21 (3.98) |  | 397.23 (5.58) | 411.45 (5.65) |  | 469.93 (6.86) | 497.26 (6.95) |  | 567.33 (8.36) | 612.45 (8.56) |  | 541.10 (8.04) | 565.39 (7.94) |
| Check-up fee | 72.02 (1.01) | 75.43 (1.04) |  | 97.46 (1.39) | | 103.92 (1.45) |  | 91.5 (1.29) | 94.89 (1.30) |  | 90.53 (1.32) | 96.16 (1.34) |  | 86.06 (1.27) | 97.70 (1.37) |  | 93.76 (1.39) | 95.54 (1.34) |
| Operation Fee | 1477.35 (20.75) | 1445.65 (19.96) |  | 1402.29 (20.00) | | 1436.62 (20.10) |  | 1412.41 (19.83) | 1411.11 (19.38) |  | 1452.67 (21.22) | 1437.04 (20.08) |  | 1370.11 (20.19) | 1414.12 (19.75) |  | 1418.29 (21.06) | 1453.81 (20.42) |
| Color ultrasound fee | 29.44 (0.41) | 32.13 (0.44) |  | 25.5 (0.36) | | 40.01 (0.56) |  | 61.55 (0.86) | 60.59 (0.83) |  | 49.33 (0.72) | 48.6 (0.68) |  | 30.69 (0.45) | 61.73 (0.86) |  | 25.97 (0.39) | 45.59 (0.64) |
| Nursing fee | 289.45 (4.07) | 314.04 (4.34) |  | 408.24 (5.82) | | 423.53 (5.93) |  | 387.76 (5.44) | 395.22 (5.43) |  | 361.97 (5.29) | 397.99 (5.56) |  | 401.89 (5.92) | 432.28 (6.04) |  | 439.06 (6.52) | 439.59 (6.17) |
| Anesthetic fee | 532.38 (7.48) | 551.11 (7.61) |  | 546.69 (7.80) | | 572.91 (8.01) |  | 604.62 (8.49) | 592.03 (8.13) |  | 586.23 (8.56) | 578.65 (8.09) |  | 571.19 (8.42) | 578.08 (8.08) |  | 625.49 (9.29) | 642.95 (9.03) |
| Other expenses | 1520.80 (21.36) | 1546.31 (21.35) |  | 1554.18 (22.17) | | 1587.67 (22.21) |  | 1531.88 (21.51) | 1550.43 (21.29) |  | 1291.20 (18.86) | 1372.29 (19.17) |  | 1258.51 (18.55) | 1262.37 (17.63) |  | 924.33 (13.73) | 1040.43 (14.61) |
| All-in cost | 7120.72 (100.00) | 7242.50 (100.00) |  | 7009.35 (100.00) | | 7148.45 (100.00) |  | 7122.94 (100.00) | 7282.95 (100.00) |  | 6846.15 (100.00) | 7156.99 (100.00) |  | 6785.18 (100.00) | 7158.92 (100.00) |  | 6734.74 (100.00) | 7119.72 (100.00) |

Supplementary Table 5 Average hospitalization cost of lacunar infarction mellitus (Yuan) and component ratio (%)

| **Expense categories** | 2016 | | |  | | 2017 | | |  | | 2018 | | |  | | 2019 | | |  | | 2020 | | |  | | 2021 | | |
| --- | --- | --- | --- | --- | --- | --- | --- | --- | --- | --- | --- | --- | --- | --- | --- | --- | --- | --- | --- | --- | --- | --- | --- | --- | --- | --- | --- | --- |
|  | Treatment  (n =29) | Control (n = 19) |  | | Treatment  (n =33) | | Control (n = 27) |  | | Treatment  (n =47) | | Control (n = 14) |  | | Treatment  (n =2) | | Control (n = 12) |  | | Treatment  (n =54) | | Control (n = 26) |  | | Treatment  (n =29) | | Control (n = 21) |  |
| Treatment cost | 357.84 (4.04) | 434.63 (5.45) |  | | 360.29 (4.06) | | 399.70 (4.93) |  | | 348.37 (3.97) | | 401.68 (4.92) |  | | 387.25 (6.25) | | 352.75 (4.53) |  | | 281.46 (5.22) | | 353.63 (4.48) |  | | 280.40 (5.14) | | 483.70 (6.28) |  |
| Drug costs | 5451.30 (61.48) | 4865.37 (61.00) |  | | 3309.36 (37.26) | | 3686.77 (45.43) |  | | 3472.32 (39.54) | | 3161.99 (38.70) |  | | 1120.59 (18.09) | | 3332.11 (42.74) |  | | 1867.36 (34.63) | | 3214.88 (40.74) |  | | 1860.54 (34.12) | | 3615.48 (46.92) |  |
| Hospitalization fee | 503.10 (5.67) | 389.47 (4.88) |  | | 558.33 (6.29) | | 390.56 (4.81) |  | | 476.60 (5.43) | | 361.79 (4.43) |  | | 327.50 (5.29) | | 357.50 (4.59) |  | | 279.63 (5.19) | | 459.62 (5.82) |  | | 268.62 (4.93) | | 367.62 (4.77) |  |
| Laboratory Fee | 334.62 (3.77) | 353.79 (4.44) |  | | 920.79 (10.37) | | 518.56 (6.39) |  | | 816.19 (9.29) | | 957.07 (11.71) |  | | 896.50 (14.47) | | 658.00 (8.44) |  | | 669.22 (12.41) | | 831.65 (10.54) |  | | 600.90 (11.02) | | 744.52 (9.66) |  |
| Check-up fee | 146.72 (1.66) | 117.00 (1.47) |  | | 193.64 (2.18) | | 164.00 (2.02) |  | | 196.85 (2.24) | | 158.14 (1.94) |  | | 162.00 (2.62) | | 147.00 (1.89) |  | | 111.00 (2.06) | | 159.54 (2.02) |  | | 115.45 (2.12) | | 138.86 (1.80) |  |
| Operation Fee | 0.00 (0.00) | 0.00 (0.00) |  | | 0.00 (0.00) | | 0.00 (0.00) |  | | 0.00 (0.00) | | 0.00 (0.00) |  | | 170 (2.74) | | 0.00 (0.00) |  | | 122.59 (2.27) | | 13.08 (0.17) |  | | 112.76 (2.07) | | 0.00 (0.00) |  |
| Diagnostic test fee | 961.24  (10.84) | 883.79 (11.08) |  | | 1754.67 (19.76) | | 1553.19 (19.14) |  | | 1647.89 (18.77) | | 1442.21 (17.65) |  | | 1372.40 (22.15) | | 1513.12 (19.41) |  | | 885.48 (16.42) | | 1168.21 (14.80) |  | | 963.92 (17.68) | | 931.02 (12.08) |  |
| CT fee | 371.48 (4.19) | 256.79 (3.22) |  | | 556.61 (6.27) | | 287.00 (3.54) |  | | 636.62 (7.25) | | 449.79 (5.51) |  | | 402.00 (6.49) | | 400.83 (5.14) |  | | 312.67 (5.80) | | 358.19 (4.54) |  | | 378.90 (6.95) | | 297.00 (3.85) |  |
| Color ultrasound fee | 205.90 (2.32) | 100.26 (1.26) |  | | 299.24 (3.37) | | 232.78 (2.87) |  | | 353.87 (4.03) | | 128.86 (1.58) |  | | 500.50 (8.08) | | 83.33 (1.07) |  | | 210.72 (3.91) | | 53.92 (0.68) |  | | 171.59 (3.15) | | 12.00 (0.16) |  |
| Nursing fee | 265.55 (3.00) | 222.84 (2.79) |  | | 412.06 (4.64) | | 392.67 (4.84) |  | | 384.64 (4.38) | | 552.43 (6.76) |  | | 306.00 (4.94) | | 333.42 (4.28) |  | | 199.33 (3.70) | | 514.31 (6.52) |  | | 200.17 (3.67) | | 346.57 (4.50) |  |
| Anesthetic fee | 13.45 (0.15) | 18.84 (0.24) |  | | 21.15 (0.24) | | 0.67 (0.01) |  | | 3.62 (0.04) | | 0.00 (0.00) |  | | 13.50 (0.22) | | 0.00 (0.00) |  | | 8.30 (0.15) | | 1.92 (0.02) |  | | 6.62 (0.12) | | 0.00 (0.00) |  |
| Other expenses | 256.19 (2.89) | 333.40 (4.18) |  | | 494.62 (5.57) | | 488.65 (6.02) |  | | 444.75 (5.07) | | 556.89 (6.82) |  | | 537.41 (8.67) | | 618.09 (7.93) |  | | 445.17 (8.26) | | 763.24 (9.67) |  | | 493.18 (9.04) | | 769.30 (9.98) |  |
| All-in cost | 8867.40 (100.00) | 7976.19 (100.00) |  | | 8880.75 (100.00) | | 8114.53 (100.00) |  | | 8781.73 (100.00) | | 8170.84 (100.00) |  | | 6195.65 (100.00) | | 7796.15 (100.00) |  | | 5392.93 (100.00) | | 7892.19 (100.00) |  | | 5453.04 (100.00) | | 7706.07 (100.00) |  |

Supplementary Table 6 The change value, change degree and contribution rate of type 2 diabetes inpatient cost structure

| **Expense categories** | 2016-2017 | | | | |  | | 2017-2018 | | | | | |  | | 2018-2019 | | | | | |  | | 2019-2020 | | | | | |  | | 2020-2021 | | | | | |  | | 2016-2021 | | | | | |  |
| --- | --- | --- | --- | --- | --- | --- | --- | --- | --- | --- | --- | --- | --- | --- | --- | --- | --- | --- | --- | --- | --- | --- | --- | --- | --- | --- | --- | --- | --- | --- | --- | --- | --- | --- | --- | --- | --- | --- | --- | --- | --- | --- | --- | --- | --- | --- |
|  | Treatment | | Control | | |  | | Treatment | | | Control | | |  | | Treatment | | | Control | | |  | | Treatment | | | Control | | |  | | Treatment | | | Control | | |  | | Treatment | | | Control | | |  |
|  | VSV | CSV | | VSV | CSV | |  | | VSV | CSV | | VSV | CSV | |  | | VSV | CSV | | VSV | CSV | |  | | VSV | CSV | | VSV | CSV | |  | | VSV | CSV | | VSV | CSV | |  | | VSV | CSV | | VSV | CSV | |
| Treatment cost | 0.07 | 0.17 | | 0.80 | 1.81 | |  | | -0.29 | 3.83 | | -1.72 | 11.49 | |  | | -0.69 | 2.26 | | 0.41 | 3.11 | |  | | 1.65 | 3.29 | | 2.20 | 5.78 | |  | | 2.09 | 16.06 | | -0.42 | 5.52 | |  | | 2.83 | 6.34 | | 1.27 | 3.83 | |
| Drug costs | -19.46 | 48.31 | | -21.25 | 48.10 | |  | | -2.43 | 32.10 | | -0.62 | 4.14 | |  | | -11.81 | 38.62 | | -3.58 | 27.17 | |  | | 16.76 | 33.46 | | 12.16 | 31.95 | |  | | -3.40 | 26.13 | | -0.03 | 0.39 | |  | | -20.34 | 45.59 | | -13.32 | 40.16 | |
| Hospitalization fee | -0.63 | 1.55 | | -0.57 | 1.29 | |  | | -1.09 | 14.40 | | 0.31 | 2.08 | |  | | -0.72 | 2.37 | | -0.33 | 2.51 | |  | | 1.49 | 2.97 | | 0.72 | 1.89 | |  | | -1.02 | 7.82 | | -0.50 | 6.57 | |  | | -1.97 | 4.42 | | -0.37 | 1.13 | |
| Laboratory Fee | 4.24 | 10.53 | | 4.48 | 10.14 | |  | | 2.17 | 28.73 | | 2.30 | 15.40 | |  | | 6.31 | 20.62 | | 3.92 | 29.78 | |  | | -3.10 | 6.18 | | -2.28 | 5.99 | |  | | 0.26 | 2.00 | | 0.36 | 4.77 | |  | | 9.89 | 22.16 | | 8.79 | 26.51 | |
| Check-up fee | 0.52 | 1.30 | | 0.28 | 0.63 | |  | | -0.45 | 5.90 | | 3.86 | 25.82 | |  | | 0.12 | 0.41 | | -0.04 | 0.33 | |  | | 0.21 | 0.42 | | 0.14 | 0.36 | |  | | -0.13 | 1.00 | | -0.14 | 1.87 | |  | | 0.28 | 0.63 | | 0.26 | 0.78 | |
| Operation Fee | -0.05 | 0.13 | | 0.00 | 0.00 | |  | | -0.02 | 0.26 | | 0.00 | 0.00 | |  | | 0.92 | 3.01 | | 0.48 | 3.64 | |  | | 0.10 | 0.19 | | 0.53 | 1.39 | |  | | 2.21 | 17.01 | | -0.27 | 3.60 | |  | | 3.16 | 7.08 | | 0.73 | 2.20 | |
| Diagnostic test fee | 8.77 | 21.77 | | 7.05 | 15.96 | |  | | 0.42 | 5.60 | | -0.89 | 5.92 | |  | | 5.28 | 17.25 | | 0.52 | 3.92 | |  | | -12.70 | 25.35 | | -9.27 | 24.37 | |  | | -1.15 | 8.81 | | 2.27 | 29.81 | |  | | 0.62 | 1.40 | | -0.32 | 0.98 | |
| CT fee | 2.70 | 6.70 | | 5.84 | 13.22 | |  | | 1.57 | 20.71 | | 0.68 | 4.55 | |  | | -0.82 | 2.68 | | -2.00 | 15.14 | |  | | -2.66 | 5.31 | | -3.78 | 9.93 | |  | | 1.22 | 9.35 | | 0.96 | 12.63 | |  | | 2.00 | 4.48 | | 1.71 | 5.15 | |
| Color ultrasound fee | 1.45 | 3.60 | | -0.27 | 0.60 | |  | | 2.05 | 27.07 | | 2.24 | 14.95 | |  | | 2.66 | 8.69 | | 0.78 | 5.95 | |  | | -6.59 | 13.16 | | -3.58 | 9.41 | |  | | 0.72 | 5.57 | | -1.74 | 22.88 | |  | | 0.29 | 0.65 | | -2.56 | 7.73 | |
| Nursing fee | 1.34 | 3.33 | | 0.91 | 2.07 | |  | | -0.85 | 11.27 | | -0.02 | 0.11 | |  | | 0.00 | 0.02 | | -0.09 | 0.67 | |  | | 0.49 | 0.99 | | -0.12 | 0.31 | |  | | -0.48 | 3.69 | | -0.11 | 1.47 | |  | | 0.50 | 1.13 | | 0.58 | 1.75 | |
| Anesthetic fee | 0.86 | 2.13 | | 1.77 | 4.00 | |  | | -0.87 | 11.53 | | -1.10 | 7.38 | |  | | -0.92 | 3.00 | | -0.55 | 4.17 | |  | | 2.02 | 4.04 | | 1.36 | 3.57 | |  | | -0.30 | 2.30 | | -0.58 | 7.65 | |  | | 0.79 | 1.77 | | 0.89 | 2.69 | |
| Other expenses | 0.19 | 0.48 | | 0.96 | 2.17 | |  | | -0.21 | 2.79 | | -1.22 | 8.16 | |  | | -0.33 | 1.09 | | 0.48 | 3.61 | |  | | 2.32 | 4.64 | | 1.93 | 5.06 | |  | | -0.03 | 0.26 | | 0.22 | 2.83 | |  | | 1.94 | 4.35 | | 2.35 | 7.10 | |
| DSV | 40.28 | | 44.18 | | |  | | 7.57 | | | 14.96 | | |  | | 30.59 | | | 13.18 | | |  | | 50.10 | | | 38.06 | | |  | | 13.02 | | | 7.61 | | |  | | 44.62 | | | 33.16 | | |  |

Supplementary Table 7 The change value, change degree and contribution rate of planned cesarean section inpatient cost structure

| **Expense categories** | 2016-2017 | | | | |  | | 2017-2018 | | | | | |  | | 2018-2019 | | | | | |  | | 2019-2020 | | | | | |  | | 2020-2021 | | | | | |  | | 2016-2021 | | | | | |  |
| --- | --- | --- | --- | --- | --- | --- | --- | --- | --- | --- | --- | --- | --- | --- | --- | --- | --- | --- | --- | --- | --- | --- | --- | --- | --- | --- | --- | --- | --- | --- | --- | --- | --- | --- | --- | --- | --- | --- | --- | --- | --- | --- | --- | --- | --- | --- |
|  | Treatment | | Control | | |  | | Treatment | | | Control | | |  | | Treatment | | | Control | | |  | | Treatment | | | Control | | |  | | Treatment | | | Control | | |  | | Treatment | | | Control | | |  |
|  | VSV | CSV | | VSV | CSV | |  | | VSV | CSV | | VSV | CSV | |  | | VSV | CSV | | VSV | CSV | |  | | VSV | CSV | | VSV | CSV | |  | | VSV | CSV | | VSV | CSV | |  | | VSV | CSV | | VSV | CSV | |
| Treatment cost | 0.24 | 2.09 | | 0.24 | 2.09 | |  | | 0.09 | 1.46 | | 0.09 | 1.46 | |  | | 0.24 | 4.3 | | 0.24 | 4.3 | |  | | -0.07 | 0.97 | | -0.07 | 0.97 | |  | | 0.05 | 0.65 | | 0.05 | 0.65 | |  | | 0.55 | 2.67 | | 0.55 | 2.67 | |
| Drug costs | -4.92 | 42.75 | | -4.92 | 42.75 | |  | | 0.92 | 14.96 | | 0.92 | 14.96 | |  | | 0.20 | 3.60 | | 0.2 | 3.6 | |  | | -0.06 | 0.83 | | -0.06 | 0.83 | |  | | 0.45 | 5.83 | | 0.45 | 5.83 | |  | | -3.41 | 16.58 | | -3.41 | 16.58 | |
| Hospitalization fee | 1.02 | 8.86 | | 1.02 | 8.86 | |  | | -0.21 | 3.41 | | -0.21 | 3.41 | |  | | -0.46 | 8.29 | | -0.46 | 8.29 | |  | | -1.61 | 22.36 | | -1.61 | 22.36 | |  | | 1.08 | 13.99 | | 1.08 | 13.99 | |  | | -0.18 | 0.88 | | -0.18 | 0.88 | |
| Laboratory Fee | 0.98 | 8.51 | | 0.98 | 8.51 | |  | | 1.67 | 27.15 | | 1.67 | 27.15 | |  | | 1.30 | 23.42 | | 1.3 | 23.42 | |  | | 1.61 | 22.36 | | 1.61 | 22.36 | |  | | -0.62 | 8.03 | | -0.62 | 8.03 | |  | | 4.94 | 24.02 | | 4.94 | 24.02 | |
| Check-up fee | 0.41 | 3.56 | | 0.41 | 3.56 | |  | | -0.15 | 2.44 | | -0.15 | 2.44 | |  | | 0.04 | 0.72 | | 0.04 | 0.72 | |  | | 0.03 | 0.42 | | 0.03 | 0.42 | |  | | -0.03 | 0.39 | | -0.03 | 0.39 | |  | | 0.30 | 1.46 | | 0.3 | 1.46 | |
| Operation Fee | 0.14 | 1.22 | | 0.14 | 1.22 | |  | | -0.72 | 11.71 | | -0.72 | 11.71 | |  | | 0.70 | 12.61 | | 0.7 | 12.61 | |  | | -0.33 | 4.58 | | -0.33 | 4.58 | |  | | 0.67 | 8.68 | | 0.67 | 8.68 | |  | | 0.46 | 2.24 | | 0.46 | 2.24 | |
| Diagnostic test fee | -0.83 | 7.21 | | -0.83 | 7.21 | |  | | -0.58 | 9.43 | | -0.58 | 9.43 | |  | | 0.17 | 3.06 | | 0.17 | 3.06 | |  | | 1.28 | 17.78 | | 1.28 | 17.78 | |  | | 0.50 | 6.48 | | 0.5 | 6.48 | |  | | 0.54 | 2.63 | | 0.54 | 2.63 | |
| Color ultrasound fee | 0.12 | 1.04 | | 0.12 | 1.04 | |  | | 0.27 | 4.39 | | 0.27 | 4.39 | |  | | -0.15 | 2.70 | | -0.15 | 2.7 | |  | | 0.18 | 2.50 | | 0.18 | 2.5 | |  | | -0.22 | 2.85 | | -0.22 | 2.85 | |  | | 0.20 | 0.97 | | 0.2 | 0.97 | |
| Nursing fee | 1.59 | 13.81 | | 1.59 | 13.81 | |  | | -0.50 | 8.13 | | -0.5 | 8.13 | |  | | 0.13 | 2.34 | | 0.13 | 2.34 | |  | | 0.48 | 6.67 | | 0.48 | 6.67 | |  | | 0.13 | 1.68 | | 0.13 | 1.68 | |  | | 1.83 | 8.90 | | 1.83 | 8.9 | |
| Anesthetic fee | 0.40 | 3.48 | | 0.4 | 3.48 | |  | | 0.12 | 1.95 | | 0.12 | 1.95 | |  | | -0.04 | 0.72 | | -0.04 | 0.72 | |  | | -0.01 | 0.14 | | -0.01 | 0.14 | |  | | 0.95 | 12.31 | | 0.95 | 12.31 | |  | | 1.42 | 6.90 | | 1.42 | 6.9 | |
| Other expenses | 0.86 | 7.47 | | 0.86 | 7.47 | |  | | -0.92 | 14.96 | | -0.92 | 14.96 | |  | | -2.12 | 38.20 | | -2.12 | 38.2 | |  | | -1.54 | 21.39 | | -1.54 | 21.39 | |  | | -3.02 | 39.12 | | -3.02 | 39.12 | |  | | -6.74 | 32.77 | | -6.74 | 32.77 | |
| DSV | 12.33 | | 11.51 | | |  | | 8.37 | | | 6.15 | | |  | | 8.77 | | | 5.55 | | |  | | 6.81 | | | 7.20 | | |  | | 11.02 | | | 7.72 | | |  | | 22.12 | | | 20.57 | | |  |

Supplementary Table 8 The change value, change degree and contribution rate of lacunar cerebral infarction inpatient cost structure

| **Expense categories** | 2016-2017 | | | | |  | | 2017-2018 | | | | | |  | | 2018-2019 | | | | | |  | | 2019-2020 | | | | | |  | | 2020-2021 | | | | | |  | | 2016-2021 | | | | | |  |
| --- | --- | --- | --- | --- | --- | --- | --- | --- | --- | --- | --- | --- | --- | --- | --- | --- | --- | --- | --- | --- | --- | --- | --- | --- | --- | --- | --- | --- | --- | --- | --- | --- | --- | --- | --- | --- | --- | --- | --- | --- | --- | --- | --- | --- | --- | --- |
|  | Treatment | | Control | | |  | | Treatment | | | Control | | |  | | Treatment | | | Control | | |  | | Treatment | | | Control | | |  | | Treatment | | | Control | | |  | | Treatment | | | Control | | |  |
|  | VSV | CSV | | VSV | CSV | |  | | VSV | CSV | | VSV | CSV | |  | | VSV | CSV | | VSV | CSV | |  | | VSV | CSV | | VSV | CSV | |  | | VSV | CSV | | VSV | CSV | |  | | VSV | CSV | | VSV | CSV | |
| Treatment cost | 0.02 | 0.04 | | -0.52 | 1.59 | |  | | -0.09 | 1.13 | | -0.01 | 0.05 | |  | | 2.28 | 5.10 | | -0.39 | 2.76 | |  | | -1.03 | 3.11 | | -0.05 | 0.31 | |  | | -0.08 | 1.23 | | 1.80 | 10.86 | |  | | 1.10 | 1.96 | | 0.83 | 2.67 | |
| Drug costs | -24.22 | 50.00 | | -15.57 | 47.51 | |  | | 2.28 | 28.64 | | -6.73 | 33.65 | |  | | -21.45 | 47.99 | | 4.04 | 28.57 | |  | | 16.54 | 50.02 | | -2.00 | 12.20 | |  | | -0.51 | 7.83 | | 6.18 | 37.27 | |  | | -27.36 | 48.63 | | -14.08 | 45.35 | |
| Hospitalization fee | 0.62 | 1.28 | | -0.07 | 0.21 | |  | | -0.86 | 10.80 | | -0.38 | 1.90 | |  | | -0.14 | 0.31 | | 0.16 | 1.13 | |  | | -0.10 | 0.30 | | 1.23 | 7.50 | |  | | -0.26 | 3.99 | | -1.05 | 6.33 | |  | | -0.74 | 1.32 | | -0.11 | 0.35 | |
| Laboratory Fee | 6.60 | 13.63 | | 1.95 | 5.95 | |  | | -1.08 | 13.57 | | 5.32 | 26.60 | |  | | 5.18 | 11.59 | | -3.27 | 23.13 | |  | | -2.06 | 6.23 | | 2.10 | 12.81 | |  | | -1.39 | 21.35 | | -0.88 | 5.31 | |  | | 7.25 | 12.89 | | 5.22 | 16.81 | |
| Check-up fee | 0.52 | 1.07 | | 0.55 | 1.68 | |  | | 0.06 | 0.75 | | -0.08 | 0.40 | |  | | 0.38 | 0.85 | | -0.05 | 0.35 | |  | | -0.56 | 1.69 | | 0.13 | 0.79 | |  | | 0.06 | 0.92 | | -0.22 | 1.33 | |  | | 0.46 | 0.82 | | 0.33 | 1.06 | |
| Operation Fee | 0.00 | 0.00 | | 0.00 | 0.00 | |  | | 0.00 | 0.00 | | 0.00 | 0.00 | |  | | 2.74 | 6.13 | | 0.00 | 0.00 | |  | | -0.47 | 1.42 | | 0.17 | 1.04 | |  | | -0.20 | 3.07 | | -0.17 | 1.03 | |  | | 2.07 | 3.68 | | 0.00 | 0.00 | |
| Diagnostic test fee | 8.92 | 18.41 | | 8.06 | 24.60 | |  | | -0.99 | 12.44 | | -1.49 | 7.45 | |  | | 3.38 | 7.56 | | 1.76 | 12.45 | |  | | -5.73 | 17.33 | | -4.61 | 28.13 | |  | | 1.26 | 19.35 | | -2.72 | 16.41 | |  | | 6.84 | 12.16 | | 1.00 | 3.22 | |
| CT fee | 2.08 | 4.29 | | 0.32 | 0.98 | |  | | 0.98 | 12.31 | | 1.97 | 9.85 | |  | | -0.76 | 1.70 | | -0.37 | 2.62 | |  | | -0.69 | 2.09 | | -0.60 | 3.66 | |  | | 1.15 | 17.67 | | -0.69 | 4.16 | |  | | 2.76 | 4.91 | | 0.63 | 2.03 | |
| Color ultrasound fee | 1.05 | 2.17 | | 1.61 | 4.91 | |  | | 0.66 | 8.29 | | -1.29 | 6.45 | |  | | 4.05 | 9.06 | | -0.51 | 3.61 | |  | | -4.17 | 12.61 | | -0.39 | 2.38 | |  | | -0.76 | 11.67 | | -0.52 | 3.14 | |  | | 0.83 | 1.48 | | -1.10 | 3.54 | |
| Nursing fee | 1.64 | 3.39 | | 2.05 | 6.27 | |  | | -0.26 | 3.27 | | 1.92 | 9.58 | |  | | 0.56 | 1.25 | | -2.48 | 17.54 | |  | | -1.24 | 3.75 | | 2.24 | 13.67 | |  | | -0.03 | 0.46 | | -2.02 | 12.18 | |  | | 0.67 | 1.19 | | 1.71 | 5.51 | |
| Anesthetic fee | 0.09 | 0.19 | | -0.23 | 0.70 | |  | | -0.20 | 2.51 | | -0.01 | 0.05 | |  | | 0.18 | 0.40 | | 0.00 | 0.00 | |  | | -0.07 | 0.21 | | 0.02 | 0.12 | |  | | -0.03 | 0.46 | | -0.02 | 0.12 | |  | | -0.03 | 0.05 | | -0.24 | 0.77 | |
| Other expenses | 2.68 | 5.53 | | 1.84 | 5.61 | |  | | -0.50 | 6.28 | | 0.80 | 4.00 | |  | | 3.60 | 8.05 | | 1.11 | 7.85 | |  | | -0.41 | 1.24 | | 2.85 | 17.39 | |  | | 0.78 | 11.98 | | 0.31 | 1.87 | |  | | 6.15 | 10.93 | | 5.80 | 18.68 | |
| DSV | 48.44 | | 32.77 | | |  | | 7.96 | | | 20.00 | | |  | | 44.70 | | | 14.14 | | |  | | 33.07 | | | 16.39 | | |  | | 6.51 | | | 16.58 | | |  | | 56.26 | | | 31.05 | | |  |

Supplementary Table 9 Inpatients' satisfaction with dimensions and each dimension of secondary indexes

| **Dimensions & Secondary indexes** | Mean value | SD |
| --- | --- | --- |
| Medical quality | 4.47 | 0.73 |
| treatment effect | 4.40 | 0.87 |
| professional technic level | 4.43 | 0.87 |
| length of stay | 4.57 | 0.66 |
| Satisfaction of medical expenses | 4.56 | 0.66 |
| expense rationality | 4.54 | 0.74 |
| reimbursement ratio | 4.59 | 0.64 |
| Satisfaction of medical expenses | 4.34 | 0.81 |
| Ease of settlement | 4.39 | 0.84 |
| service attitude | 4.30 | 0.94 |
| Overall satisfaction | 4.46 | 0.69 |

Supplementary Table 10 Analysis of in-patient awareness of payment for single disease

| **Projects** | N | Constituent ratio (%) |
| --- | --- | --- |
| Knowledge levels |  |  |
| Not knowledgeable at all | 2 | 1.10 |
| Slightly knowledgeable | 18 | 9.94 |
| Moderately knowledgeable | 24 | 13.26 |
| Very knowledgeable | 52 | 28.73 |
| Extremely knowledgeable | 85 | 46.96 |
| Knowledge approach |  |  |
| Medical staff and hospital publicity | 164 | 90.60 |
| Internet, TV and other media | 32 | 17.70 |
| Others (friends, family, etc.) | 33 | 18.20 |
| Never ever heard | 10 | 5.50 |

Supplementary Table 11 Medical staffs' satisfaction with dimensions and each dimension of secondary indexes

| **Dimensions & Secondary Indexes** | Mean value | SD |
| --- | --- | --- |
| SDP quality | 4.07 | 0.87 |
| Number of diseases | 4.04 | 0.97 |
| Cost of diseases | 4.04 | 0.97 |
| Entry exit mechanism | 4.11 | 0.88 |
| Medical quality | 3.80 | 0.77 |
| the choice of drug | 3.75 | 1.12 |
| Diagnosis and treatment habits | 3.58 | 1.17 |
| Service and efficacy | 3.28 | 1.37 |
| length of stay | 4.22 | 0.86 |
| diagnosis and treatment behavior | 4.18 | 0.83 |
| New technology and projects | 3.42 | 1.28 |
| New treatment plan | 3.40 | 1.30 |
| advanced technology | 3.43 | 1.28 |
| doctor-patient relationship | 3.91 | 0.80 |
| the satisfaction of patients | 4.09 | 0.83 |
| doctor-patient communication | 4.06 | 0.95 |
| Efficiency and revenue | 4.08 | 0.87 |
| income | 3.79 | 1.11 |
| incentive system | 3.72 | 1.04 |
| Workload and efficiency | 4.23 | 0.83 |
| Overall satisfaction | 3.86 | 0.68 |

Supplementary Table 12 Analysis of medical staff awareness of payment for single disease

| **Projects** | N | Constituent ratio (%) |
| --- | --- | --- |
| Knowledge levels |  |  |
| Not knowledgeable at all | 1 | 0.73 |
| Slightly knowledgeable | 3 | 2.19 |
| Moderately knowledgeable | 34 | 24.82 |
| Very knowledgeable | 47 | 34.31 |
| Extremely knowledgeable | 52 | 37.95 |
| Knowledge approach |  |  |
| Hospital publicity | 71 | 95.60 |
| Government and industry information policy release | 47 | 59.10 |
| Never ever heard | 19 | 1.50 |

Supplementary Table 13 Problems in the implementation of SDP

| **Projects** | N | Constituent ratio (%) |
| --- | --- | --- |
| The coverage of diseases is insufficient | 99.00 | 72.30 |
| The awareness and acceptance rate of patients is low | 92.00 | 67.20 |
| The implementation cost is high and the hospital income is reduced | 56.00 | 40.90 |
| High rate of disease variation | 68.00 | 49.60 |
| The management process is tedious and increases the workload | 48.00 | 35.00 |
| The clinical pathway is not reasonable | 34.00 | 24.80 |

# Questionnaire survey

## Supplementary File 1 The inpatients’ satisfaction on the implement of SDP Questionnaire (in the original languages and translated)

尊敬的病员朋友及家属：

您好！为了解“单病种付费”实施情况，能更好地为患者提供优质的服务，特向您了解有关情况，恳请您利用几分钟时间，填写这份问卷，并提出宝贵的意见，感谢您的支持，祝早日康复！

Dear inpatients and your family:

We would appreciate your valuable feedback on the implementation of single disease payment (SDP), aiming to further enhance the provision of high-quality services for patients. Please kindly spare a few moments to complete this questionnaire and provide your valuable feedback. We sincerely appreciate a swift recovery!

• 您在本次住院期间是否执行了“单病种付费”？A.是

B.否 (无需完成7-14题)

• Do you have a private health care coverage? A.YES

B.NO (No need to complete questions 7-14)

| **序号**  **NO.** | **调查内容**  **Investigation Item** | **选项或反馈**  **Options or feedback** |
| --- | --- | --- |
| 1 | 您入住的科室是？  Which department did you check in? | A.内科 B.外科 C.妇产科 D.儿科 E.急诊科 F.ICU G.其他  A. Internal medicine B. Surgery C. Obstetrics and Gynecology D. Pediatrics E. Emergency Department F.IUC G. other Department |
| 2 | 您的医保类别是？  Which category is your insurance category? | A.福建省医保 B.福州市医保 C.异地医保 D.无医保 E.其他  A. Fujian Province Medical insurance B. Fuzhou Medical insurance C. Remote medical insurance D. No medical insurance E. other |
| 3 | 您对“单病种付费”是否了解？  Have you ever known about SDP? | A.非常了解 B.比较了解 C.一般了解 D.不太了解 E.完全不了解A. Not knowledgeable at all B. Slightly knowledgeable C. Moderately knowledgeable D. Very knowledgeable E. Extremely knowledgeable |
| 4 | 您是通过以下哪种途径了解到“单病种付费”？  In which of the following ways did you learn about the SDP? | A.医务人员及医院宣传 B.网络、电视等其他媒体 C.他人（朋友、家人等）D.没有听说过  A. Medical staff and hospital publicity B. Internet, TV and other media C. Others (friends, family, etc.) D. Never ever heard |
| 5 | 您认为实行“单病种付费”能够规范诊疗行为吗？  Do you think the SDP can standardize the diagnosis and treatment behavior? | A.非常同意 B.比较同意 C.一般同意 D.不太同意 E.完全不同意  A. Strongly Agree B. Agree C. Undecided D. Disagree E. Strongly Disagree |
| 6 | 您认为实行“单病种付费”能够控制医疗费用，减轻患者负担吗？  Do you think the implementation of SDP can control medical costs and reduce the burden of patients? | A.非常同意 B.比较同意 C.一般同意 D.不太同意 E.完全不同意  A. Strongly Agree B. Agree C. Undecided D. Disagree E. Strongly Disagree |
| 7 | 入院时，主管医生或护士是否向您告知“单病种付费”相关内容？  Did the doctor or nurse in charge inform you about the SDP on admission? | A.非常同意 B.比较同意 C.一般同意 D.不太同意 E.完全不同意  A. Strongly Agree B. Agree C. Undecided D. Disagree E. Strongly Disagree |
| 8 | 您对本次实行“单病种付费”的医务人员专业技术水平是否满意？  Are you satisfied with the professional level of the medical staff in this SDP policy? | A.非常同意 B.比较同意 C.一般同意 D.不太同意 E.完全不同意  A. Strongly Agree B. Agree C. Undecided D. Disagree E. Strongly Disagree |
| 9 | 您对本次实行“单病种付费”后的治疗效果是否满意？  Are you satisfied with the treatment effect after the implementation of SDP? | A.非常同意 B.比较同意 C.一般同意 D.不太同意 E.完全不同意  A. Strongly Agree B. Agree C. Undecided D. Disagree E. Strongly Disagree |
| 10 | 您对本次实行“单病种付费”的住院天数是否满意？  Are you satisfied with the length of hospital stay under the SDP policy? | A.非常同意 B.比较同意 C.一般同意 D.不太同意 E.完全不同意  A. Strongly Agree B. Agree C. Undecided D. Disagree E. Strongly Disagree |
| 11 | 您对本次实行“单病种付费”后的医疗费用合理性是否满意？  Are you satisfied with the rationality of the medical expenses after the implementation of SDP this time? | A.非常同意 B.比较同意 C.一般同意 D.不太同意 E.完全不同意  A. Strongly Agree B. Agree C. Undecided D. Disagree E. Strongly Disagree |
| 12 | 您对本次实行“单病种付费”后的医保报销比例是否满意？  Are you satisfied with the reimbursement ratio of medical insurance after the implementation of SDP this time? | A.非常同意 B.比较同意 C.一般同意 D.不太同意 E.完全不同意  A. Strongly Agree B. Agree C. Undecided D. Disagree E. Strongly Disagree |
| 13 | 您对本次实行“单病种付费”的费用结算便捷性是否满意？  Are you satisfied with the convenience of SDP this time? | A.非常同意 B.比较同意 C.一般同意 D.不太同意 E.完全不同意  A. Strongly Agree B. Agree C. Undecided D. Disagree E. Strongly Disagree |
| 14 | 您对本次实行“单病种付费”的医务人员服务态度是否满意？  Are you satisfied with the service attitude of the medical staff in this SDP policy? | A.非常同意 B.比较同意 C.一般同意 D.不太同意 E.完全不同意  A. Strongly Agree B. Agree C. Undecided D. Disagree E. Strongly Disagree |
| 15 | 您对目前的“单病种付费”政策有什么建议？  What are your suggestions on the SDP policy? | 建议：  Suggestions: |

## Supplementary File 2 The medical staffs’ satisfaction on the implement of SDP Questionnaire (in the original languages and translated)

尊敬的各位医护工作者：

您好！为了解“单病种付费”实施情况，以期发现问题并提出对策，同时为今后向更加精细化的支付方式过渡奠定基础，特向您了解有关情况，恳请您利用几分钟时间，填写这份问卷，并提出宝贵的意见，感谢您的支持，祝工作顺利！

Dear inpatients and your family:

We would appreciate your valuable feedback on the implementation of single disease payment (SDP), aiming to identify problems and put forward measures while also laying a foundation for future transitions towards more refined payment methods. Please kindly spare a few moments to complete this questionnaire and provide your valuable feedback. We sincerely anticipate the smoothy work to you!

| **序号**  **NO.** | **调查内容**  **Investigation Item** | **选项或反馈**  **Options or feedback** |
| --- | --- | --- |
| 1 | 您的年龄是？  What’s your age? | A.20-30岁 B.30-40岁 C.40-50岁 D.50-60岁 E.60岁以上 F.其他  A.20-30 years old B.30-40 years old C.40-50 years old D.50-60 years old E. Over 60 years old F. Other |
| 2 | 您的职称是？  What's your professional title? | A.初级 B.中级 C.高级 D.其他  A. Primary B. Middle C. High D. Other |
| 3 | 您的工作岗位是？  What is your position? | A.医师 B.护理 C.医技 D.其他  A. Physician B. Nurse C. Technician D. Other |
| 4 | 您的学历是？  What is your education background? | A.大专及以下 B.本科 C.硕士及以上 D.其他  A. College education or below b. bachelor degree C. Master degree or above D. Other |
| 5 | 您对“单病种付费”是否了解？  Have you ever known about SDP? | A.非常了解 B.比较了解 C.一般了解 D.不太了解 E.完全不了解A. Not knowledgeable at all B. Slightly knowledgeable C. Moderately knowledgeable D. Very knowledgeable E. Extremely knowledgeable |
| 6 | 您是通过以下哪种途径了解到“单病种付费”？  In which of the following ways did you learn about the SDP? | A.医务人员及医院宣传 B.网络、电视等其他媒体 C.他人（朋友、家人等）D.没有听说过  A. Medical staff and hospital publicity B. Internet, TV and other media C. Others (friends, family, etc.) D. Never ever heard |
| 7 | 医院或科室经常组织关于“单病种付费”的培训吗？  Does the hospital or department often organize training on SDP? | A.非常同意 B.比较同意 C.一般同意 D.不太同意 E.完全不同意  A. Strongly Agree B. Agree C. Undecided D. Disagree E. Strongly Disagree |
| 8 | 您认为目前“单病种付费”病种的覆盖面的广泛程度？  What is the current coverage of SDP? | A.非常同意 B.比较同意 C.一般同意 D.不太同意 E.完全不同意  A. Strongly Agree B. Agree C. Undecided D. Disagree E. Strongly Disagree |
| 9 | 您认为目前“单病种付费”的费用限额标准合理程度？  How reasonable do you think the current fee limit for SDP is? | A.非常同意 B.比较同意 C.一般同意 D.不太同意 E.完全不同意  A. Strongly Agree B. Agree C. Undecided D. Disagree E. Strongly Disagree |
| 10 | 您认为“单病种付费”的临床路径合理程度？  How reasonable is the clinical pathway of SDP? | A.非常同意 B.比较同意 C.一般同意 D.不太同意 E.完全不同意  A. Strongly Agree B. Agree C. Undecided D. Disagree E. Strongly Disagree |
| 11 | 您认为目前“单病种付费”的进入-退出机制的合理程度？  How reasonable do you think the current SDP entry-exit mechanism is? | A.非常同意 B.比较同意 C.一般同意 D.不太同意 E.完全不同意  A. Strongly Agree B. Agree C. Undecided D. Disagree E. Strongly Disagree |
| 12 | 您对“单病种付费”对规范诊疗行为，提高医疗质量的效果满意吗？  Are you satisfied with the impact of SDP on standardizing medical behavior and enhancing medical quality? | A.非常同意 B.比较同意 C.一般同意 D.不太同意 E.完全不同意  A. Strongly Agree B. Agree C. Undecided D. Disagree E. Strongly Disagree |
| 13 | 您对“单病种付费”对临床医师用药选择的变化满意吗？  Are you satisfied with how the SDP has changed clinicians' medication choices? | A.非常同意 B.比较同意 C.一般同意 D.不太同意 E.完全不同意  A. Strongly Agree B. Agree C. Undecided D. Disagree E. Strongly Disagree |
| 14 | 您对“单病种付费”对临床医师诊疗习惯的变化满意吗？  Are you satisfied with the changes of clinicians' diagnosis and treatment habits caused by the SDP? | A.非常同意 B.比较同意 C.一般同意 D.不太同意 E.完全不同意  A. Strongly Agree B. Agree C. Undecided D. Disagree E. Strongly Disagree |
| 15 | 您对“单病种付费”带来的对患者诊疗效果的影响满意吗？  Are you satisfied with the effect of SDP on the diagnosis and treatment of patients? | A.非常同意 B.比较同意 C.一般同意 D.不太同意 E.完全不同意  A. Strongly Agree B. Agree C. Undecided D. Disagree E. Strongly Disagree |
| 16 | 您对“单病种付费”带来的工作量和效率的变化满意吗？  Are you satisfied with the changes in workload and efficiency brought about by the SDP? | A.非常同意 B.比较同意 C.一般同意 D.不太同意 E.完全不同意  A. Strongly Agree B. Agree C. Undecided D. Disagree E. Strongly Disagree |
| 17 | 您对“单病种付费”对探索新诊疗方案的影响满意吗？  Are you satisfied with the impact of SDP on the exploration of new diagnosis and treatment options? | A.非常同意 B.比较同意 C.一般同意 D.不太同意 E.完全不同意  A. Strongly Agree B. Agree C. Undecided D. Disagree E. Strongly Disagree |
| 18 | 您对“单病种付费”对先进医疗技术应用的影响满意吗？  Are you satisfied with the impact of SDP on the use of advanced medical technologies? | A.非常同意 B.比较同意 C.一般同意 D.不太同意 E.完全不同意  A. Strongly Agree B. Agree C. Undecided D. Disagree E. Strongly Disagree |
| 19 | 您对于目前实行“单病种付费”给您的收入带来的影响满意吗？  Are you satisfied with the impact of the current SDP on your income? | A.非常同意 B.比较同意 C.一般同意 D.不太同意 E.完全不同意  A. Strongly Agree B. Agree C. Undecided D. Disagree E. Strongly Disagree |
| 20 | 您对当前“单病种付费”的激励机制满意吗？  Are you satisfied with the current incentive mechanism of SDP? | A.非常同意 B.比较同意 C.一般同意 D.不太同意 E.完全不同意  A. Strongly Agree B. Agree C. Undecided D. Disagree E. Strongly Disagree |
| 21 | 您对“单病种付费”对住院天数的影响满意吗？  Are you satisfied with | A.非常同意 B.比较同意 C.一般同意 D.不太同意 E.完全不同意  A. Strongly Agree B. Agree C. Undecided D. Disagree E. Strongly Disagree |
| 22 | 您认为患者对实行“单病种付费”的认可度或满意度如何？  Are you satisfied with the impact of SDP on the length of stay? | A.非常同意 B.比较同意 C.一般同意 D.不太同意 E.完全不同意  A. Strongly Agree B. Agree C. Undecided D. Disagree E. Strongly Disagree |
| 23 | 您对“单病种付费”对医患关系的影响满意吗？  Are you satisfied with the impact of SDP on the physician-patient relationship? | A.非常同意 B.比较同意 C.一般同意 D.不太同意 E.完全不同意  A. Strongly Agree B. Agree C. Undecided D. Disagree E. Strongly Disagree |
| 24 | 您支持实行“单病种付费”吗？  Do you support with the implement of SDP? | A.非常同意 B.比较同意 C.一般同意 D.不太同意 E.完全不同意  A. Strongly Agree B. Agree C. Undecided D. Disagree E. Strongly Disagree |
| 25 | 您认为目前实行“单病种付费”存在的困难有哪些？  What do you think are the current difficulties in the implementation of SDP? | A.病种覆盖面不足 B.患者知晓率低，接受度不高  C.实施成本高，医院收益降低 D.疾病变异率高  E.管理过程繁琐，增加工作量 F.临床路径不合理 G.其他  A. inadequate coverage diseases B. low awareness and acceptance C. Implementation cost is high, the hospital income reduce D. disease high mutation rate E. Tedious management process increase work F. Unreasonable clinical pathway G. Others |
| 26 | 您对目前的“单病种付费”政策有什么建议？  What are your suggestions on the SDP policy? | 建议：  Suggestions: |
